# Fast and accurate branch lengths estimation for phylogenomic trees

**DOI:** 10.1186/s12859-015-0821-8

**Published:** 2016-01-07

**Authors:** Manuel Binet, Olivier Gascuel, Celine Scornavacca, Emmanuel J. P. Douzery, Fabio Pardi

**Affiliations:** Laboratoire d’Informatique de Robotique et de Microélectronique de Montpellier (LIRMM), CNRS, Université de Montpellier, Montpellier, France; Institut de Biologie Computationnelle, Montpellier, France; Institut des Sciences de l’Evolution de Montpellier, CNRS, IRD, EPHE, Université de Montpellier, France

**Keywords:** Phylogenomics, Supertree, Branch lengths, Gene rates, Distance-based, Least-squares

## Abstract

**Background:**

Branch lengths are an important attribute of phylogenetic trees, providing essential information for many studies in evolutionary biology. Yet, part of the current methodology to reconstruct a phylogeny from genomic information — namely supertree methods — focuses on the topology or structure of the phylogenetic tree, rather than the evolutionary divergences associated to it. Moreover, accurate methods to estimate branch lengths — typically based on probabilistic analysis of a concatenated alignment — are limited by large demands in memory and computing time, and may become impractical when the data sets are too large.

**Results:**

Here, we present a novel phylogenomic distance-based method, named ERaBLE (Evolutionary Rates and Branch Length Estimation), to estimate the branch lengths of a given reference topology, and the relative evolutionary rates of the genes employed in the analysis. ERaBLE uses as input data a potentially very large collection of distance matrices, where each matrix is obtained from a different genomic region — either directly from its sequence alignment, or indirectly from a gene tree inferred from the alignment. Our experiments show that ERaBLE is very fast and fairly accurate when compared to other possible approaches for the same tasks. Specifically, it efficiently and accurately deals with large data sets, such as the OrthoMaM v8 database, composed of 6,953 exons from up to 40 mammals.

**Conclusions:**

ERaBLE may be used as a complement to supertree methods — or it may provide an efficient alternative to maximum likelihood analysis of concatenated alignments — to estimate branch lengths from phylogenomic data sets.

**Electronic supplementary material:**

The online version of this article (doi:10.1186/s12859-015-0821-8) contains supplementary material, which is available to authorized users.

## Background

With the continuous growth of genome sequencing capabilities, phylogenetic inference is increasingly based on large collections of genomic regions, if not entire genomes [1–3]. We have entered the era of phylogenomics — the study of evolution at a genomic scale.

New methodological challenges arise in this field. Clearly, the large amount of data — sequences from several taxa and large collections of genes — makes computational efficiency essential. Besides quantity, the nature of the data is also a concern, and it is extremely important to correctly account for the distinctive features of a typical phylogenomic data set: for example the heterogeneity in the evolution of genomic regions [4–9], and the fact that each region is typically sequenced in a subset of the taxa under analysis, with only partial overlap between different subsets [10, 11].

In this paper, we focus on the problem of how to efficiently and accurately estimate the branch lengths of a tree in a phylogenomic context, a problem for which, to date, only computationally-intensive techniques appear to be available. Yet, evolutionary distance information is essential to answer several important biological questions, from molecular dating [12, 13] of events such as speciations, to the reconciliation of gene trees with a species tree [14], or to the measure of biodiversity in conservation biology [15]. Another goal here is the efficient estimation of the relative rates of evolution of different genomic regions. This information – strictly linked to branch lengths – is also very useful, for example to recognize the diverse selective pressures acting on different parts of the genome [16, 17]. Tree inferences in a phylogenomic context fall roughly into three frameworks: the supertree, the superalignment and the medium-level framework. We consider them in relation to our goals of branch length and gene rate estimation.

*Supertree* approaches [18, 19] combine the information from several phylogenetic trees into a larger phylogeny. A strength of these methods is that the source trees can come from different types of data, such as DNA or protein sequences, or even morphological data. In a phylogenomic context, each source phylogeny is inferred from a different locus, with standard methods such as maximum likelihood, maximum parsimony or distance-based approaches. Within this category, MRP (Matrix Representation with Parsimony) [20, 21] and its derived methods (e.g., SuperFine [22]) are to date the most widely used approaches. In its standard form, MRP does not use branch length information in the source trees (if present), a limitation that is shared by most supertree methods — with very few exceptions, such as BWD (Build with Distances) [23], ACS (Average Consensus Supertree) [24] and SDM (Super Distance Matrix) [25]. As a consequence, virtually all supertree approaches are unable to provide meaningful estimates for branch lengths (MRP may provide branch weights, but these should be interpreted as a measure of evidence, not evolutionary change), or any estimate at all for gene rates.

*Superalignment* methods are the other classical approach for phylogenomic tree inference. They concatenate all available genomic sequence alignments into a unique alignment (often called a *character supermatrix*), which is then analyzed with standard or specially-tailored phylogenetic reconstruction algorithms [26]. These methods — whose accuracy relies on the use of state-of-the-art statistical inference techniques (typically maximum likelihood or Bayesian methods) — naturally model branch lengths and across-site rate heterogeneity. However they are computationally demanding, and may become impractical if computing time or memory are limited, or when the concatenated alignment is very large. Moreover, heterogeneity in the evolutionary processes at different genomic regions — which is readily handled in a supertree context — may require the use of models such as partition models [8, 9] or mixture models [6, 7]. These models, however, further increase the number of parameters to estimate, and consequently computational costs.

Lastly, the *medium-level* [25, 27, 28] framework combines the information originating from the different loci at a level that is intermediate between sequence alignments and complete gene trees. For example, this intermediate level may consist of partial trees — such as quartets [29, 30] — or pairwise distances between gene sequences [24, 25]. Specifically, distance-based methods naturally account for and can estimate branch lengths, and in some cases they can even estimate gene rates [5, 25]. Moreover, they are relatively light computationally. The method we present here, ERaBLE (Evolutionary Rates and Branch Length Estimation), falls within this category. Unlike other phylogenomic methods, however, its goal is not tree estimation, and ERaBLE should be used to complement existing approaches that do not estimate branch lengths and/or gene rates. Alternatively, it can be used on its own when the evolutionary relationships among the species under consideration are largely known.

Note that distance-based phylogenomic methods such as ERaBLE, ACS [24] and SDM [25] can be used both in the medium-level framework – when the input distances are directly estimated from genomic alignments – but also in the supertree framework – when the input distances require prior inference of a collection of gene trees. We will see examples of this in our experiments (“Results and discussion” section).

The methodology we propose here can be seen as a generalization of classical weighted least squares (WLS) branch length estimation, to the case where multiple distance matrices are estimated from different genomic regions. In fact if only one matrix is given, ERaBLE behaves exactly like WLS. WLS fits the branch lengths of a tree *T* so as to make the distances between its leaves as close as possible to the input distances. Formally, it minimises the criterion $\sum _{i<j}w_{\textit {ij}}\left (\delta _{\textit {ij}}-d_{\textit {ij}}^{T}\right)^{2}$, where the *δ*_*ij*_ denote the input distances, the $d^{T}_{\textit {ij}}$ are the distances between the leaves of *T* (determined by the lengths assigned to its branches), and the weights *w*_*ij*_>0 express the confidence in the estimate *δ*_*ij*_. When multiple distance matrices are provided, we face the problem that, due to rate heterogeneity among the alignments, their distances cannot readily be compared to $d_{\textit {ij}}^{T}$. ERaBLE thus applies a rescaling of the input distances, in order to use them for branch length estimation. Compared to WLS, this entails surprisingly little computational overhead.

In the following, we first describe our new method and the data sets on which we compared its performance to that of other possible approaches for the same task (“Methods” section). Then, we present the results of our experiments on these data sets (“Results and discussion” section).

## Methods

In this paper, we assume that the analysis focuses on a collection of orthologous genomic regions, or genes, *G*_1_,*G*_2_,…,*G*_*m*_, whose evolution mostly differs because of rate heterogeneity. In other words, the trees describing their evolution are topologically compatible [31]. This is an optimal scenario for the methods we describe here, but it does not preclude their application to real-world datasets where this assumption will be necessarily violated to some degree. Gene tree topological incompatibilities may in fact arise due to incomplete lineage sorting [32, 33], gene duplication and loss [34], or even lateral gene transfer (see [35] for an excellent review of these phenomena). An even stronger assumption, which is useful to clarify the meaning of branch lengths and rates at a genomic level, is that of the *proportional model* [4, 36], which we describe further below.

### Defining phylogenomic branch lengths

The length of branch *e* in the gene tree for *G*_*k*_, denoted here $b_{e}^{(k)}$, generally represents the average (or expected) number of substitutions per site, occurred between the endpoints of *e*. If we let *x* and *y* denote these endpoints, we can rewrite this as:

(1) $$ b_{e}^{(k)}=\frac{s^{(k)}_{xy}}{N_{k}},   $$

where $s^{(k)}_{\textit {xy}}$ is the (expected) number of substitutions in *G*_*k*_ occurred between *x* and *y*, and *N*_*k*_ is the sequence length of gene *G*_*k*_.

We wish to give the same meaning to the branch lengths of the phylogenomic (or species) tree representing the evolution of genes *G*_1_,*G*_2_,…,*G*_*m*_. If we define the length of branch *e* in this tree as the average (or expected) number of substitutions per site between its endpoints *x* and *y*, we then have:

(2) $$ b_{e}= \frac{\sum\limits_{k=1}^{m}s_{xy}^{(k)}}{\sum\limits_{k=1}^{m}N_{k}}.   $$

This definition determines the relationship between the branch lengths in the species tree and those in the gene trees. If we let $N= \sum _{k=1}^{m}N_{k}$, and use Eq. (1), then Eq. (2) can be rewritten as:

(3) $$ b_{e} = \frac{1}{N}\sum_{k=1}^{m}N_{k}b_{e}^{(k)}   $$

In other words, branch length *b*_*e*_ in the species tree is equal to an average of the corresponding branch lengths $b_{e}^{(k)}$ in the gene trees, weighted by the lengths of the gene sequences.

Note that in this paper we assume that genes are sampled in different, partially overlapping sets of taxa, meaning that a branch in a gene tree will in general correspond to a path in the species tree. Thus, in Eq. (3), and in the equations that follow, it is more accurate to interpret *b*_*e*_ and $b_{e}^{(k)}$ as lengths of paths connecting the same nodes across all trees, depending on the taxa sampled for each gene.

### The proportional model

In order to provide a stronger link among branch lengths in gene trees and in the species tree, and to set a meaningful scale for the gene rate estimates, we now introduce the *proportional model* [4, 36], an implicit assumption of many phylogenomics methods [4, 5, 36], including ours. This model assumes that each gene *G*_*k*_ induces the same tree up to a multiplicative constant for branch lengths, *r*_*k*_, representing its evolutionary rate (and up to removal of branches leading to taxa for which *G*_*k*_ is not sampled). In other words, if we let $b_{e}^{(k)}$ denote the length of a branch *e* (or a path, see above) in the gene tree for *G*_*k*_, then

(4) $$ \frac{b_{e}^{(k)}}{r_{k}} \text{is constant for all}~k = 1, \ldots, m.   $$

This model is a rough approximation of biological reality, as typically the relative values of the gene rates *r*_1_,*r*_2_,…,*r*_*m*_ may vary over time — a phenomenon known as heterotachy [37]. Nevertheless, this simple model greatly restricts the number of parameters to estimate and leads to robust analyses.

The proportional model, as specified by Eq. (4), defines *relative* rates, that is, it determines *r*_*k*_ up to a multiplicative constant. Here, we take *r*_*k*_ as the rate of *G*_*k*_, relative to the “phylogenomic rate”, that is, we require $r_{k} = b_{e}^{(k)}/{b_{e}}$. Equation (3) then implies that the weighted average of gene rates must be 1. In fact, by substituting $b_{e}^{(k)}$ with *r*_*k*_*b*_*e*_ into Eq. (3), and dividing both sides by *b*_*e*_, we obtain:

(5) $$ \frac{1}{N}\sum_{k=1}^{m}N_{k}r_{k}=1.   $$

We will use this equation later on, to set a meaningful scale for the gene rates output by our method (and others). The same rescaling will be applied to the returned branch lengths, as they are strictly linked to the rates.

### The ERaBLE method

The new method presented here, ERaBLE (*Evolutionary Rates and Branch Length Estimation*), simultaneously estimates gene rates and the branch lengths of a phylogenomic tree of given topology, using a collection of distance matrices — one distance matrix per gene *G*_*k*_. As we illustrate in our experiments (*Results* section), these distance matrices can either be directly estimated from pairwise alignments of the gene sequences, or they can be calculated from gene trees inferred for each *G*_*k*_. A C++ implementation of ERaBLE is available on the web at http://www.atgc-montpellier.fr/erable/.

Let *L*_*k*_ designate the set of taxa for which the sequence of *G*_*k*_ is available. For *i,j*∈*L*_*k*_, let then $\delta _{\textit {ij}}^{(k)}$ denote the input distance for gene *G*_*k*_ between taxa *i* and *j*. Given a tree topology $\mathcal {T}$ with leaves labelled by the taxa in $L=\bigcup _{k=1}^{m} L_{k}$, the goal is to estimate the branch lengths of $\mathcal {T}$ and the evolutionary rates of the *m* genes under consideration. $\mathcal {T}$ can either reflect a well-known phylogeny for the taxa in *L*, or it can be inferred prior to ERaBLE’s execution, for example using MRP or other supertree methods. We do not make any assumption on the degree of overlap between the taxon sets *L*_*k*_. Extremely sparse data sets may not determine a unique optimal solution to our estimation problem, but this does not prevent the application of ERaBLE.

Now let $\hat {b}_{e}$ denote the estimated length for branch *e*. This determines the *additive distance*$\widehat {d}_{\textit {ij}}$ between any two taxa *i* and *j*, simply defined as the sum of the $\hat {b}_{e}$ for all *e* in the path between *i* and *j* in $\mathcal {T}$. For mathematical convenience, we choose to estimate the inverses of gene rates: we refer to $\hat {\alpha }_{k}$, the estimate for 1/*r*_*k*_, as the *scale factor* of gene *G*_*k*_. ERaBLE thus seeks the values of $\hat {b}_{e}$, for all branches in $\mathcal {T}$, and of $\hat {\alpha }_{k}$, for *k*=1,2,…,*m*, that solve the following optimization problem:

(6) $${} \left \{ \begin{array}{ll} \text{minimize} & Q(\hat{\alpha},\hat{b})=\sum\limits_{k=1}^{m}\sum\limits_{\{i,j\} \subset L_{k}}w^{(k)}_{ij}\left(\hat{\alpha}_{k}\delta_{ij}^{(k)}- \widehat{d}_{ij}\right)^{2}, \\ \text{subject to} & \sum\limits_{k=1}^{m} {Z}_{k} \hat{\alpha}_{k} = \sum\limits_{k=1}^{m} {Z}_{k}. \end{array} \right.  $$

ERaBLE can efficiently solve this problem for any choice of positive values for $w^{(k)}_{\textit {ij}}$ and *Z*_*k*_. Below, we explain the rationale behind the objective function $Q(\hat {\alpha },\hat {b})$ and the constraint in problem (6), and provide practical choices for $w^{(k)}_{\textit {ij}}$ and *Z*_*k*_. Then, we briefly describe the algorithm that allows ERaBLE to efficiently solve problem (6). Details are provided in Additional file 1. Lastly, we show how to rescale the optimal values for $\hat {b}_{e}$ and $\hat {\alpha }_{k}$, so that they comply with their definitions in Eqs. (3) and (5).

***The objective function.*** As predicted by the proportional model, we would like the distances in the phylogenomic tree to be approximately equal to the gene-specific distances, up to the multiplicative factor *r*_*k*_. Thus, we would like to set the $\hat {b}_{e}$ and $\hat {\alpha }_{k}$, so that:

$${} \hat{d}_{ij} \approx \frac{\delta_{ij}^{(k)}}{r_{k}} \approx \hat{\alpha}_{k}\delta_{ij}^{(k)} \text{\quad for all \(k\in\{1,2, \ldots, m\}\) and \({i,j} \in L_{k}\)}. $$

The optimisation criterion $Q(\hat {\alpha },\hat {b})$ provides a score for the discrepancy between the $\hat {d}_{\textit {ij}}$ and the scaled distances $\hat {\alpha }_{k}\delta _{\textit {ij}}^{(k)}$. It is a WLS criterion, where $w_{\textit {ij}}^{(k)}$ is a strictly positive weight indicating the confidence given to the distance estimate $\delta _{\textit {ij}}^{(k)}$, and which ideally is inversely proportional to its variance. In our experiments, we have chosen the simple approach of setting $w_{\textit {ij}}^{(k)} = N_{k}$ (i.e., the length of the alignment for gene *G*_*k*_), but ERaBLE is capable of using more sophisticated weightings (e.g., [5, 38]).

WLS is a special case of GLS, a class of criteria that account for the covariances between the $\delta _{\textit {ij}}^{(k)}$. However, GLS criteria are rarely used for phylogenetic inference, because of the computational complexity of optimizing them, and because of the difficulty of evaluating the covariances. WLS is a good compromise, and it is notably used in the well-known algorithm of Fitch and Margoliash [39] and in FastME [40].

Criterion $Q(\hat {\alpha },\hat {b})$ is similar to those by Bevan et al. [5] and Criscuolo et al. [25]. The optimisation problems in these papers, however, seek optimal values for $\hat {d}_{\textit {ij}}$ directly, without assuming any relationship between these distances and a tree (namely without assuming additivity). ERaBLE, instead, assumes a particular topology $\mathcal {T}$, and constrains the distances $\hat {d}_{\textit {ij}}$ to be additive with respect to $\mathcal {T}$, meaning that its problem unknowns are the branch lengths in $\mathcal {T}$.

***The constraint.***$Q(\hat {\alpha },\hat {b})$ is trivially minimized by setting all $\hat {\alpha }_{k}=0$, and all $\hat {b}_{e}=0$. In order to obtain more meaningful solutions, while ensuring mathematical tractability, we adopt a linear constraint over the $\hat {\alpha }_{k}$: the constraint in (6) is in fact the most general form for such a linear constraint. In Additional file 1, we show that the right-hand side in this constraint is irrelevant to the end results, as it only determines their scale, which is subsequently reset by the step described in *Rescaling the outputs* below.

As to the choice for *Z*_*k*_, the two simplest approaches are to set *Z*_*k*_=1 [4, 25] or *Z*_*k*_=*N*_*k*_. The latter results in a constraint that is similar in spirit to Eq. (5) above, as it constrains more strongly the rates (or more precisely their inverses) of long genes. However, our experiments have shown that both these approaches can incur in significant over-estimation of the scale factors $\hat {\alpha }_{k}$ for genes appearing in a small subset *L*_*k*_ of closely related taxa. In Additional file 2, we show a small example where the reasons for this are evident. In order to deal with this problem, we have chosen to set $Z_{k}= N_{k} \sum _{\text {\textit {i,j}} \in L_{k}} \delta _{\textit {ij}}^{(k)}$ in all the experiments below, an approach that at the same time puts a stronger constraint on the scale factors of long genes – like (5) above – and that we have experimentally verified to largely fix the over-estimation problem for the $\hat {\alpha }_{k}$.

***Solving the problem.*** The one in (6) is a classic quadratic programming problem, which can be solved using Lagrange multipliers [41]. As we show in Additional file 1, this yields a system of $\mathcal {O}(n+m)$ linear equations in $\mathcal {O}(n+m)$ unknowns (all the $\hat {b}_{e}$ and the $\hat {\alpha }_{k}$), where *n* is the number of taxa in *L*, and *m* is the number of genes. Calculating naïvely the coefficients of this system and solving it would require $\mathcal {O}\!\left (mn^{4} + (n + m)^{3}\right)$ time and $\mathcal {O}\!\left ((n+m)^{2}\right)$ auxiliary memory (i.e., not including the memory to store the input), but careful adaptation of techniques for WLS branch length calculation [5, 42, 43] leads to a reduction of the algorithm’s complexity to $\mathcal {O}\left (mn^{2} + n^{3}\right)$ time and $\mathcal {O}\left (mn+n^{2}\right)$ auxiliary memory. In Additional file 1, we describe this algorithm in detail.

Given that problem (6) can be seen as a generalization, for several distance matrices, of standard WLS branch length estimation, it is interesting to note that, for $m=\mathcal {O}(n)$, their computational complexities coincide — as standard WLS requires $\mathcal {O}\left (n^{3}\right)$ time and $\mathcal {O}\left (n^{2}\right)$ memory [42]. If instead *m*>>*n*, which is the most common scenario in phylogenomics, an attractive aspect of ERaBLE is that its complexity grows linearly in *m*, which makes it particularly suited to analyze phylogenomic data sets from large collections of genes (typically several thousands) sampled across a moderate number of taxa (few hundreds at most). This is indeed the scenario that we have tested in the experiments in the “Results and discussion” section, where *m* varies from 500 to about 7,000 and *n*=40.

Finally, we remark that for some data sets the optimal solution of problem (6) may not be unique. This can happen when some pairs of taxa do not co-occur in any input distance matrix (note that this is a necessary but not sufficient condition for multiplicity of solutions). All such cases are recognized by ERaBLE, and the user is notified of the existence of multiple alternative solutions beyond the one returned.

***Rescaling the outputs.*** Equation (5) shows that, as a consequence of their definition, the gene rates should have a weighted average of 1. We thus require that the estimated rates also satisfy this property, meaning that we need to rescale the $\hat {\alpha }_{k}$ so that the inverses of the new scale factors satisfy Eq. (5). In other words, we multiply the $\hat {\alpha }_{k}$ obtained by solving problem (6) by a *correction factorc* such that

$$ \frac{1}{N}\sum_{k=1}^{m}\frac{N_{k}}{c \cdot \hat{\alpha}_{k}}=1 $$

By solving this equation for *c*, we obtain:

(7) $$ c = \frac{1}{N} \sum_{k=1}^{m}\frac{N_{k}}{\hat{\alpha}_{k}}   $$

Moreover, note that in order for $\hat {d}_{\textit {ij}} \approx c\cdot \hat {\alpha }_{k}\delta _{\textit {ij}}^{(k)}$ to still hold, the same rescaling by *c* must be applied to the estimated branch lengths. In conclusion, ERaBLE returns:

$$\frac{1}{c \cdot \hat{\alpha}_{k}} \quad \text{and }\quad c\cdot\hat{b}_{e} $$

as estimates of *r*_*k*_ and *b*_*e*_ — the rate of gene *G*_*k*_ and the phylogenomic length of branch *e*, respectively.

### Other phylogenomic distance-based methods

In our experiments, we have compared ERaBLE to a number of other approaches that bioinformaticians and evolutionary biologists may adopt in order to estimate gene rates and the branch lengths of a species tree in a phylogenomic context. These approaches are implemented as analysis pipelines, and described in detail in the “Results and discussion” section. While some of these pipelines implement standard techniques such as maximum-likelihood or distance-based analysis of a concatenated alignment, most pipelines are based on two phylogenomic distance-based methods that we now describe.

***SDM*** (Super Distance Matrix) has the objective to construct a distance matrix summarizing the topological signal in a collection of gene-specific distance matrices [25]. This “average” matrix can then be used to infer a phylogenomic tree, using distance-based methods based on a single matrix. SDM applies two transformations to the input matrices — it multiplies each of them by a scale factor, and adds a scalar to each column and row (thus extending or shrinking external branches in the underlying gene tree) — with the goal of bringing them as close as possible to each other. The matrices thus obtained are then averaged to obtain a matrix that can then be analyzed with other distance-based methods. Our experiments use SDM^*^, a variant of SDM that only applies the scale factor transformation to the input matrices, which avoids altering the ratio between the lengths of internal and external branches in the reconstructed tree. We note that the implementation of SDM^*^ includes a preprocessing step that corrects the input matrices to make them satisfy the triangle inequality. Since this step, as expected, affected negatively the estimation of branch lengths (but helps that of the tree topology), we removed it from the original code. In our experiments, the average matrix produced by SDM^*^ is used to estimate the branch lengths of a fixed topology $\mathcal {T}$ using standard OLS, and gene rate estimates are obtained by taking the inverses of the scale factors returned by SDM^*^. Average distances and scale factors are rescaled as described for ERaBLE, that is, multiplied by the correction factor *c* in Eq. (7) above.

***DistR*** [5] was conceived to estimate gene rates from a collection of distance matrices, and from the alignments used to calculate the distances. DistR uses the alignments to approximate the variances of the input distances, with the classical formulae by Bulmer [38]. These variances are then used in a distance-based optimization problem akin to that solved by SDM^*^ — the main difference being the constraint on the scale of the results. DistR returns estimates for the gene rates, and, as a byproduct, a distance matrix that we use to estimate the branch lengths of a fixed topology $\mathcal {T}$ using standard OLS, as done for SDM^*^. No rescaling of the outputs was conducted for DistR, as it automatically produces rates and distances at a meaningful scale.

### Data sets

In this section, we describe the data sets that we have used in our experiments to evaluate the performance of ERaBLE and competing methods. The first data set consists of 500 simulated replicates: for each replicate, we take a random tree over 40 taxa, and for each tree we simulate sequence data for 500 genes, which are only present in a random subset of taxa, and evolve at different rates. The second data set consists of the 6,953 exon alignments for 40 mammals in OrthoMaM v8 [44]. Detailed descriptions follow.

***Simulated data.*** Each of the 500 replicates is obtained as follows.

- *Gene trees.* A tree *T*^0^ is taken randomly (without replacement) from the 5,000 trees on *n*=40 taxa in the original test data set for PhyML [45]. This tree is then rescaled to a total branch length of 1, by dividing all branch lengths by their sum. Call the resulting tree *T*^1^. We then construct *m*=500 gene trees *T*_1_,…,*T*_*m*_ by multiplying the lengths of all the branches in *T*^1^ by factors *t*_1_,…,*t*_*m*_ randomly drawn from a continuous uniform distribution on the interval [0.4,9]. This interval gives biologically realistic branch lengths [45].
- *Sequence generation.* For each gene tree *T*_*k*_, we generate a DNA alignment consisting of *n*=40 sequences of length *N*_*k*_, where *N*_*k*_ is an integer drawn uniformly from the interval [200,600]. We chose relatively short sequences to avoid making the simulated data sets too informative, so as to be able to discriminate among the estimation accuracies of the methods tested. Each alignment is generated with Seq-Gen [46], using *T*_*k*_ and the model K2P +*Γ*, with ratio between transition and transversion rates *R*=2 (equivalent to *κ*=4 ([47] Sec. 1.2.4)) and with a continuous gamma distribution with shape parameter 1, to model rate heterogeneity across sites.
- *Missing data.* To simulate the partial overlap in the gene presence/absence patterns typical of real data sets, for each alignment we randomly remove a number of sequences. More precisely, for each of the *m* alignments generated in the previous step, we draw a parameter *p* uniformly between 0 and 1, and then we suppress each sequence with probability *p*. If the number of remaining sequences in *L*_*k*_ is less than 4, then we leave 4 sequences chosen randomly out of the 40, so as to guarantee a minimum amount of data to estimate the rate for that gene.
- *Model tree definition.* We call the tree that we wish to reconstruct the “model tree”, and we denote it by *T*. Clearly, *T* must be the same as *T*^0^ and *T*^1^, up to their scale, and up to the removal of the taxa missing from all the simulated alignments. In order to define the correct scale of the model tree, we define tree *T*^2^, with the same topology as *T*^0^ and *T*^1^, and branch lengths defined by $b_{e}= \frac {1}{N} \sum _{k=1}^{m} N_{k}b_{e}^{(k)}$, where $b_{e}^{(k)}$ denotes the length of *e* in *T*_*k*_, and $N=\sum _{k=1}^{m} N_{k}$. Note that this is the same as Eq. (3), whose justification is amply given above. Finally, we obtain the model tree *T* by taking the restriction of *T*^2^ on the set of taxa $L=\bigcup _{k=1}^{m}L_{k}$.
- *Model rates definition.* Similarly to the model tree, the “model gene rates” must be the same as *t*_1_,…,*t*_*m*_ up to their scale. The absolute values of *t*_1_,…,*t*_*m*_ are in fact unrecoverable from the data. By imposing Eq. (5) to the rescaled rates, we must have:
  $$ r_{k}=\frac{t_{k}}{F} \text{, \quad where}~F=\frac{1}{N} \sum_{k=1}^{m}N_{k}t_{k}. $$

***OrthoMaM data set.*** OrthoMaM (v8) [44] consists of a collection of single-copy orthologous phylogenetic markers, selected among the genomes of the 40 mammals in the Ensembl v73 database [48]. We downloaded the entire set of the 6,953 nucleotide exon alignments in OrthoMaM v8, filtered with trimAl [49]. Alignment lengths *N*_*k*_ range from 231 to 17,103 (median: 702), and each alignment contains a variable subset *L*_*k*_ of taxa, with 4≤|*L*_*k*_|≤40 (median: 27).

## Results and discussion

In order to compare the performance of ERaBLE to that of other approaches, we have conducted a number of experiments on the data sets described in the “Methods” section. For each of the 500 simulated replicates and for the OrthoMaM data set, we compare the branch length and gene rate estimates obtained by a number of competing approaches, including ERaBLE. For the OrthoMaM data set (6,953 genes), which is an order of magnitude larger than the simulated replicates (500 genes), we also compare their running times and memory usage.

Since, to the best of our knowledge, no tool is readily available for the simultaneous estimation of branch lengths and gene rates in a phylogenomic context, for our comparisons we have assembled a number of pipelines from existing methods. Besides ERaBLE, these methods include SDM^*^ [25] and DistR [5], which however were conceived for other tasks than ours. We refer to the “Methods” for a brief description of how we adapted these tools to our goals. We describe the pipelines below.

### Analysis protocol

The OrthoMaM data set and each replicate in the simulated data set have the same structure: they consist of *m* gene alignments *A*_1_,*A*_2_,…,*A*_*m*_ over the taxon sets *L*_1_,*L*_2_,…,*L*_*m*_ (*m*=500 for the simulated data sets, *m*=6,953 for OrthoMaM). In addition to these inputs, the tested methods are also provided with a reference topology $\mathcal {T}$, over the set of taxa $L= \bigcup _{k=1}^{m}L_{k}$, to which they aim to assign branch lengths. For simulated data, $\mathcal {T}$ is the topology of the model tree *T*, whereas for OrthoMaM $\mathcal {T}$ is the mammalian tree topology in Additional file 5. The outputs are a tree estimate $\hat {T}$ with topology $\mathcal {T}$, and gene rate estimates $\hat {r}_{1},\ldots,\hat {r}_{m}$.

**Fig. 1:**
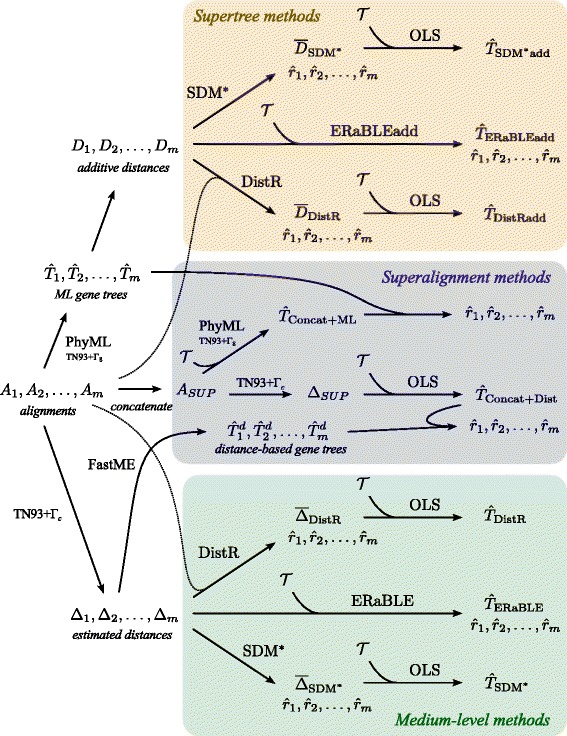
Pipelines of the analyses applied to both data sets, represented as flowcharts. We refer to the “Analysis protocol” subsection for a detailed description of each analysis method

The tested methods are classified in the three frameworks described in the “Background” section: supertree, superalignment and medium-level. Note that for distance estimation, as well as for maximum likelihood (ML) tree reconstruction, we use the model TN93+ *Γ*, as it is the most complex nucleotide substitution model for which an analytic formula for pairwise distance estimation is available. In the following, we denote by *Γ*_*c*_ the continuous Gamma distribution used for pairwise distance estimation, and by *Γ*_8_ the discrete Gamma distribution based on 8 categories, which we adopt for ML tree inference. Also note that for pairwise distance estimation, the shape parameter for the Gamma distribution cannot be estimated from the data, and thus must be set to a realistic value by the user [47] (more detail on this point below). All tested methods are depicted schematically in Fig. 1 and their names together with short descriptions can be found in Table 1. A detailed description follows.

**Table 1:** Names and short descriptions of the methods tested

Name	Brief description
Concat+Dist	Distance-based analysis of the concatenated alignment
Concat+ML	ML analysis of the concatenated alignment
SDM^*^add	SDM^*^ run on the gene tree distance matrices (+ post-processing)
DistRadd	DistR run on the gene tree distance matrices (+ post-processing)
ERaBLEadd	ERaBLE run on the gene tree distance matrices
SDM^*^	SDM^*^ run on the estimated distance matrices (+ post-processing)
DistR	DistR run on the estimated distance matrices (+ post-processing)
ERaBLE	ERaBLE run on the estimated distance matrices

***Supertree methods.*** For each alignment *A*_*k*_ we infer a gene tree $\hat {T}_{k}$ with PhyML [45, 50], using the model TN93+ *Γ*_8_. The shape parameter for the Gamma distribution is set to 1 for the simulated data sets (that is, the value used to generate the data), and left free to estimate for the OrthoMaM data set. Unless otherwise stated, in the following experiments PhyML is free to estimate the topology of $\hat {T}_{k}$, which is realistic when gene trees are inferred as part of a separate analysis, for example to provide the input for supertree topology reconstruction. In other experiments, we have constrained PhyML to reconstruct gene trees of topology agreeing with $\mathcal {T}$, an approach that significantly reduces running times. (More precisely, the topology of $\hat {T}_{k}$ is constrained to be the restriction of $\mathcal {T}$ to *L*_*k*_.) This is the correct way to proceed when the only goal is the estimation of branch lengths in a reference tree. We will come back on this second approach when comparing the computational efficiencies of the methods tested.

Standard supertree methods, such as MRP [20, 21], would then only consider the topologies of the inferred gene trees $\hat {T}_{1},\hat {T}_{2},\ldots,\hat {T}_{m}$, but this makes it impossible to estimate branch lengths for the phylogenomic tree. In order to conserve branch length information, we construct the additive distance matrices *D*_1_,*D*_2_,…,*D*_*m*_ corresponding to these gene trees — that is, the distance between taxa *i* and *j* in *D*_*k*_ equals the sum of the lengths of the branches between *i* and *j* in $\hat {T}_{k}$. Note that, as additive distances uniquely determine a tree [51], *D*_*k*_ can just be interpreted as a different representation for $\hat {T}_{k}$. We test three methods based on these additive matrices (hence “add” in their names).

- SDM^*^add. We run SDM^*^ on *D*_1_,*D*_2_,…,*D*_*m*_, with *D*_*k*_ weighted by the alignment length *N*_*k*_. The average matrix and scale factors thus obtained are then multiplied by the scaling factor *c* in Eq. (7), thus giving a scaled average matrix $\overline {D}_{\text {SDM}^{*}}$, and gene rate estimates (the inverses of the resulting scale factors). Finally, on the basis of $\overline {D}_{\text {SDM}^{*}}$ we assign OLS branch lengths to the reference topology $\mathcal {T}$, using FastME [40].
- *DistRadd*. We run DistR on *D*_1_,*D*_2_,…,*D*_*m*_(and *A*_1_,*A*_2_,…,*A*_*m*_), thus obtaining gene rate estimates and an average matrix $\overline {D}_{\text {DistR}}$. The latter is then used to assign OLS branch lengths to $\mathcal {T}$, with FastME.
- *ERaBLEadd*. We run ERaBLE on *D*_1_,*D*_2_,…,*D*_*m*_ and $\mathcal {T}$, with the weighings for $w_{\textit {ij}}^{(k)}$ and *Z*_*k*_ described in the “Methods” section. ERaBLE directly provides gene rate estimates and branch length estimates for $\mathcal {T}$.

Note that it is problematic to evaluate the variances of the distances computed by SDM^*^ and DistR (those in $\overline {D}_{\text {SDM}^{*}}$ and $\overline {D}_{\text {DistR}}$, respectively). This is why we used OLS branch length estimation for the last step in SDM^*^add and DistRadd.

***Medium-level methods.*** From each alignment *A*_*k*_, we estimate a distance matrix *Δ*_*k*_, using FastME [40] with the model TN93+ *Γ*_*c*_. Note that estimation of the shape parameter for the Gamma distribution would require joint comparison of multiple sequences [47], but here we only use pairwise comparisons. Thus, we set the shape parameter to 1 for the simulated data sets (that is, the value used to generate the data), and to 0.5 for the OrthoMaM data set, as we consider this as a realistic estimate for mammals. (E.g., the median shape parameter estimated by PhyML when inferring the OrthoMaM gene trees is 0.493.) We test three methods identical to those described above for supertree methods, except that they use the estimated matrices *Δ*_1_,*Δ*_2_,…,*Δ*_*m*_ instead of the additive matrices deriving from the ML gene trees. We call these methods SDM^*^, *DistR* and *ERaBLE*. (See again Fig. 1.)

***Superalignment methods.*** Let *A*_*SUP*_ denote the alignment obtained by concatenating *A*_1_,…,*A*_*m*_. We test two methods based on *A*_*SUP*_.

- *Concat+ML*. We assign branch lengths to the reference topology $\mathcal {T}$ by running topology-constrained PhyML on *A*_*SUP*_, with the model TN93+ *Γ*_8_. We call the resulting tree $\hat {T}_{Concat+ML}$. Here the shape parameter for the Gamma distribution is left free to estimate. In fact, even though for each gene alignment *A*_*k*_ taken separately we may set this parameter to 1 for the simulated data, or to 0.5 for OrthoMaM, these values cannot be used on the concatenation *A*_*SUP*_. This is because the alignments *A*_1_,*A*_2_,…,*A*_*m*_ derive from trees at different scales, meaning that rate variation in *A*_*SUP*_ will be larger than that on a single *A*_*k*_, and the shape parameters smaller (PhyML estimates 0.487 for OrthoMaM, and 0.7 on average for the simulated data). As to gene rate estimates, $\hat {r}_{k}$ is then obtained as the ratio between the total length of the ML gene tree $\hat {T}_{k}$ (a source tree for supertree methods) and the total length of the tree that is obtained from $\hat {T}_{Concat+ML}$ by taking its restriction to *L*_*k*_. For OrthoMaM, which, unlike the simulated data set, does not have model gene rates and a model tree, we take the outputs of this method as reference. The choice of PhyML over more computationally efficient alternatives is due to its greater availability of models, which may entail better accuracy. (See also Additional file 7, where we report about the effects of using alternative ML methods in our experiments.)
- *Concat+Dist*. From *A*_*SUP*_, we estimate a distance matrix *Δ*_*SUP*_, using FastME with the model TN93+ *Γ*_*c*_. The shape parameter for the Gamma distribution is set to the value estimated above by PhyML on *A*_*SUP*_. Then, on the basis of *Δ*_*SUP*_, we assign OLS branch lengths to the reference topology $\mathcal {T}$, using FastME. Call the resulting tree $\hat {T}_{Concat+Dist}$. Finally, in order to estimate gene rates, we use the same procedure as that for Concat+ML, but in a distance-based context: $\hat {r}_{k}$ is obtained as the ratio between the total length of a distance-based gene tree $\hat {T}^{d}_{k}$ and the length of the restriction of $\hat {T}_{Concat+Dist}$ to *L*_*k*_. Distance-based gene trees $\hat {T}^{d}_{1},\ldots,\hat {T}^{d}_{m}$ are obtained from the estimated distance matrices *Δ*_1_,…,*Δ*_*m*_ using FastME with default options.

### Results and discussion for the simulated data

**Fig. 2:**
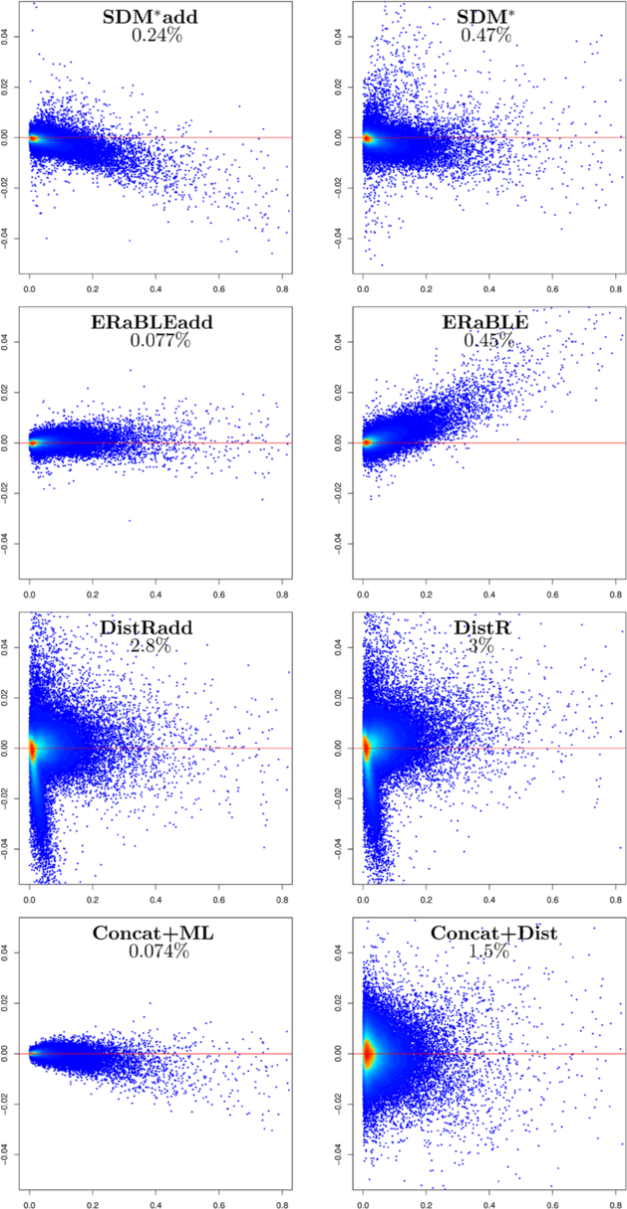
Accuracy of branch length estimates in the simulated data set. For each method, model branch lengths *b*_*e*_ (x-axis) are plotted against estimation errors $\hat {b}_{e} - b_{e}$ (y-axis) for all branches in all 500 model trees (500×77=38,500 points per plot). Colors (from blue to red) indicate increased density of points. The horizontal red line corresponds to no estimation error. Method names are shown at the top of each plot, followed by the mean (over 500 values) of the fraction of variance unexplained of (*b*_*e*_) relative to $(\hat {b}_{e})$ (see Additional file 3)

**Fig. 3:**
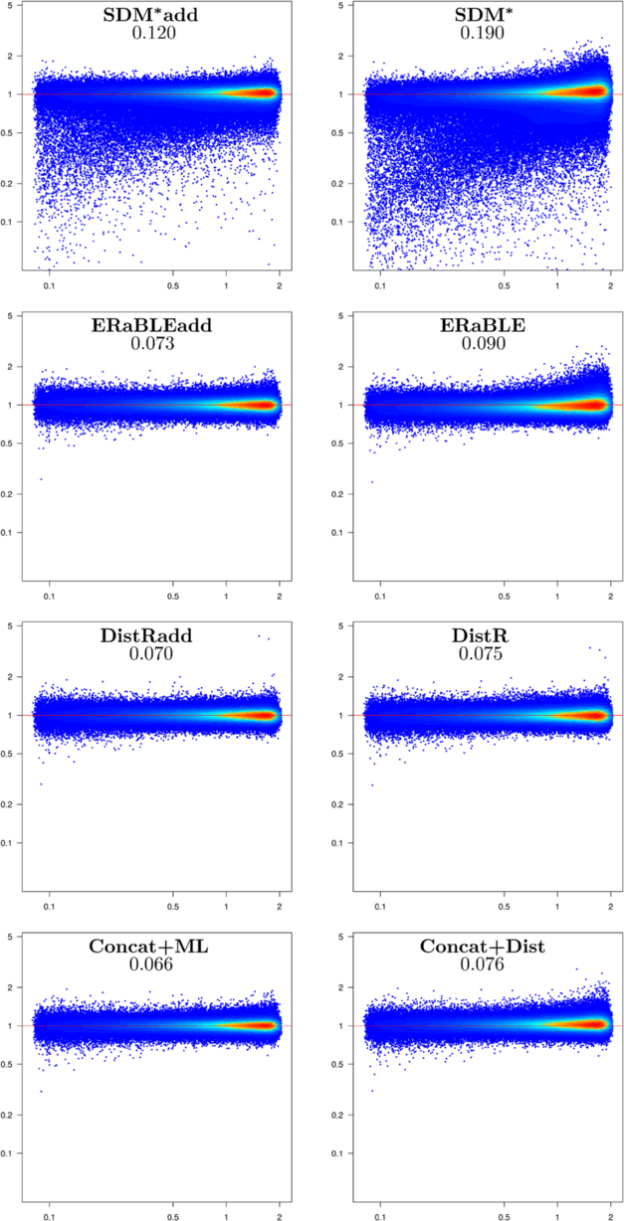
Estimation accuracy for gene rates in the simulated data set. Log-log scatterplots showing model gene rates *r*_*k*_ (x-axis) against error ratios $\hat {r}_{k}/r_{k}$ (y-axis) for all genes in all 500 replicates (500 × 500=250,000 points per plot). Note that errors are measured with ratios, instead of differences. Colors (from blue to red) indicate increased density of points. The horizontal red line corresponds to no estimation error. Method names are shown at the top of each plot, followed by the mean absolute log-ratio between estimated and model gene rates (see Additional file 3)

Given the large number of replicates, the simulated data set is especially useful to compare the estimation accuracy of the methods tested. For each method we have plotted estimation errors against the correct values of the parameters to estimate (branch lengths and gene rates), which are known for the simulated data. Figures 2 and 3 show the accuracies of all tested methods in the estimation of branch lengths and gene rates, respectively. For gene rates, the scatterplots are logarithmic, as rates are inherently ratios (e.g. rates *x* and 1/*x*, with *x*>0, should be depicted as equally distant from rate 1).

***Branch length estimation.*** The most accurate estimates of branch lengths are produced by Concat+ML and ERaBLEadd (see Fig. 2). Then, intermediate results are obtained by SDM^*^add, ERaBLE and SDM^*^, where some biases are observed: SDM^*^add seems to underestimate branch lengths, whereas ERaBLE appears to overestimate them, proportionally to the branch length. Currently, we do not have an explanation for these biases, which however are small (≤5 *%*) relative to the length of a branch (cf. the slope of the point cloud). Finally, DistRadd, DistR and Concat+Dist are all affected by relatively strong estimation problems for branch lengths: consider, for example, the mean fraction of variance unexplained, which for these methods is tens of times that of Concat+ML and ERaBLEadd. For DistR this is not surprising, as this method was only conceived to estimate gene rates (and not for species tree estimation) [5]. As for Concat+Dist, it is clear that the construction of a distance matrix from the superalignment entails a significant loss of information.

***Gene rate estimation.*** With the exception of SDM-based methods (SDM^*^ and SDM^*^add), all methods are approximately equally accurate in the estimation of gene rates (see Fig. 3), the best method being, as expected, Concat+ML. As apparent in the two scatterplots at the top of Fig. 3, SDM-based methods often strongly underestimate (by a factor of 2 or more) the rates of some genes. Typically these are genes that are only present in a small subset of closely related taxa. Moreover, for the other genes where this problem is not present, rate estimates tend to be slightly overestimated (see the red core of the point cloud, which lies *above* the horizontal red line). The reasons for this issue lie in the constraint used in the optimization problem solved by SDM^*^, which causes the same phenomena as those described in Additional file 2. The constraint used by ERaBLE avoids these issues.

***Discussion.*** A common feature of the experiments on branch length and gene rate estimation above is that medium-level methods are generally less accurate than their supertree counterparts (compare SDM^*^ to SDM^*^add, ERaBLE to ERaBLEadd, DistR to DistRadd). Again, this is not surprising, as supertree methods are based on additive distance matrices (*D*_1_,*D*_2_,…,*D*_*m*_), which are expected to be more accurate estimates of the correct distances than the distances estimated directly from the alignments (*Δ*_1_,*Δ*_2_,…,*Δ*_*m*_). However, inferring additive distance matrices comes at a (computational) cost, as we shall show on the experiments on the OrthoMaM data set in the next section.

We conclude noting that the only methods that do not incur in any major accuracy problem on the simulated data set are ERaBLE, ERaBLEadd and Concat+ML. However, their running times and memory requirements are very different: on this data set, Concat+ML is five hundred times slower than ERaBLE (about 3 h 30 m vs. 25 s on average for a single replicate on a cluster machine with 200 GB RAM and 2.66 GHz CPU) and requires far more memory (4.2 GB vs. 70 MB). In this case the computational effort to analyse the simulated data sets is clearly not problematic for Concat+ML. This is because a simulated replicate data set is relatively small (*m*=500). We look in more detail at running times and memory usage in the experiments in the next section, which are on a computationally more challenging data set.

### Results and discussion for the OrthoMaM data set

Assessing estimation accuracy on the OrthoMaM data set is more problematic than on the simulated data set, first, because the correct values for the branch lengths and gene rates are not known and, second, because statistical noise may play an important role here, as no replicates are available. We address the former issue by adopting the estimates obtained by Concat+ML as reference values. This is justified by the observation that Concat+ML provides the most accurate branch length and gene rate estimates on the simulated data set.

On the other hand, the OrthoMaM data set allows us to observe the robustness of the methods tested to violations of the proportional model, whose assumptions are not expected to hold in real data sets. Moreover, given the relatively large number of genes, this phylogenomic data set is particularly appropriate to assess the computational feasibility of the approaches we implemented.

***Computational efficiencies.*** Running times and memory usages of the tested methods are reported in Table 2. We decomposed running times in two parts: first (*T*_1_), we look at the times necessary for preprocessing steps (essentially gene tree estimation for supertree methods and distance estimation for medium-level methods); second (*T*_2_), we show the remaining running times, to actually produce branch length and gene rate estimates.

**Table 2:** Computational efficiencies on the OrthoMaM data set for the tested methods

	Concat+Dist	Concat+ML	SDM^*^add	DistRadd	ERaBLEadd	SDM^*^	DistR	ERaBLE
*T*_1_	≈0	3 h 20 m/39 h 28 m				2 m 46 s	2 m 46 s	2 m 46 s
*T*_2_	5 m 41 s	41 h 16 m	8 h 2 m	2 h 9 m	7 s	8 h 33 m	2 h 6 m	7 s
*M*	889 MB	117 GB	1.2 GB	2.8 GB	222 MB	1.2 GB	3.0 GB	221 MB

Note.— The first row gives (*T*_1_) the running time to obtain the data on which subsequent computations are based: the superalignment and the distance-based gene trees for Concat+Dist, the superalignment and ML gene trees for Concat+ML, the ML gene trees and resulting additive distances for the three supertree methods, and the estimated distances for the three medium-level methods. When ML gene trees are used (Concat+ML, SDM*add, DistRadd and ERaBLEadd), two alternative approaches are possible and therefore two running times are provided: first that to infer trees with fixed topology (3 h 20 m), and then that to infer trees where the topology is also estimated (39 h 28 m). The second row gives (*T*_2_) the remaining running time to obtain estimates for branch lengths and gene rates. The third row (**M**) gives the maximum amount of memory allocated. All the experiments were conducted on a PC with 4 GB RAM and a 2.7 GHz CPU, except branch length estimation (*T*_2_ and **M**) for Concat+ML, which, because of the large memory requirements, was run on a cluster machine with 200 GB RAM and a 2.66 GHz CPU

Preprocessing times (*T*_1_ in Table 2) show an advantage of medium-level methods (*T*_1_ in the order of the min), over supertree methods and Concat+ML, and the advantage of constraining PhyML to only optimize branch lengths and model parameters (*T*_1_ in h), rather than also seeking an ML topology (*T*_1_ in days). Note however that running times in preprocessing steps is highly and easily parallelizable, meaning that waiting times on parallel architectures will be much lower than the running times indicated here.

Actual processing times (*T*_2_) and memory requirements (**M**) in Table 2 illustrate the main strength of the new methods we propose here: while for most methods the running times are in the order of the hours (up to about 41 h for branch length estimation in Concat+ML) and memory usage in the order of the gigabytes, ERaBLE and ERaBLEadd only require a few seconds and a few hundred megabytes on the OrthoMaM data set. Particularly heavy are the memory requirements for Concat+ML: only users with access to large memory machines may use this method on a large data set (with several thousands of genes) such as OrthoMaM. As for the difference between ERaBLE and the other distance-based methods (SDM-based and DistR-based), this is consistent with the differences in computational complexities of these methods, which only for ERaBLE is linear in *m*. The only method with computational costs comparable to those of ERaBLE is Concat+Dist, which however on the simulated data leads to inaccurate branch length estimates.

**Fig. 4:**
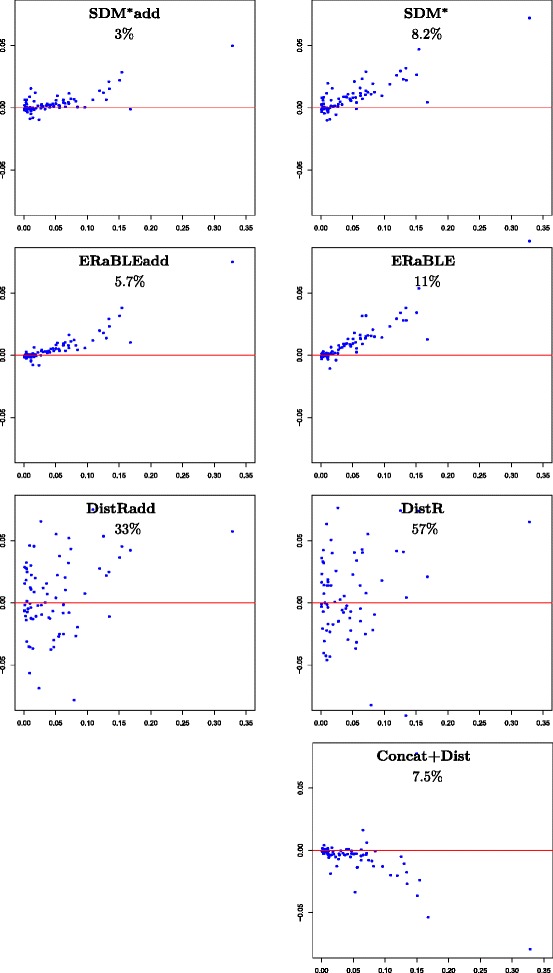
Accuracy of branch length estimates in the OrthoMaM data set. For each method, the 77 branch lengths $\hat {b}^{ML}_{e}$ estimated by Concat+ML (x-axis) are plotted against the differences $\hat {b}_{e} - \hat {b}^{ML}_{e}$ (y-axis) (where $\hat {b}_{e}$ is the estimate for the length of *e* obtained by the method at the top of the plot). The horizontal red line corresponds to no difference between the two estimates. Method names are shown at the top of each plot, followed by the fraction of variance unexplained of $(\hat {b}^{ML}_{e})$ relative to $(\hat {b}_{e})$ (see Additional file 3)

***Branch length estimation.*** Figure 4 shows the accuracy of all tested methods in the estimation of branch lengths. These experiments confirm that, not surprisingly, DistR-based methods are inaccurate at this task — as already observed in the simulated data set. Moreover, it is clear that the tested methods provide branch length estimates at slightly different scales, as their scatterplots tend to be distributed along non-horizontal lines. SDM-based and ERaBLE-based methods produce branches that are on average 5–20 % longer than those estimated by Concat+ML (the same holds for DistR-based methods, although it is harder to observe, because of the large variance of the estimates), whereas Concat+Dist tends to produce shorter branches.

The main reason for these discrepancies is the presence in OrthoMaM of an inverse correlation between the rate of a gene and the depth of its alignment: whereas superalignment methods are sensitive to gene alignment depths – with branch lengths estimates more influenced by genes with many aligned sequences, and thus evolving less rapidly – this is not true for the other tested methods. This observation explains the scale differences observed, as we explain in more detail in Additional file 4.

**Fig. 5:**
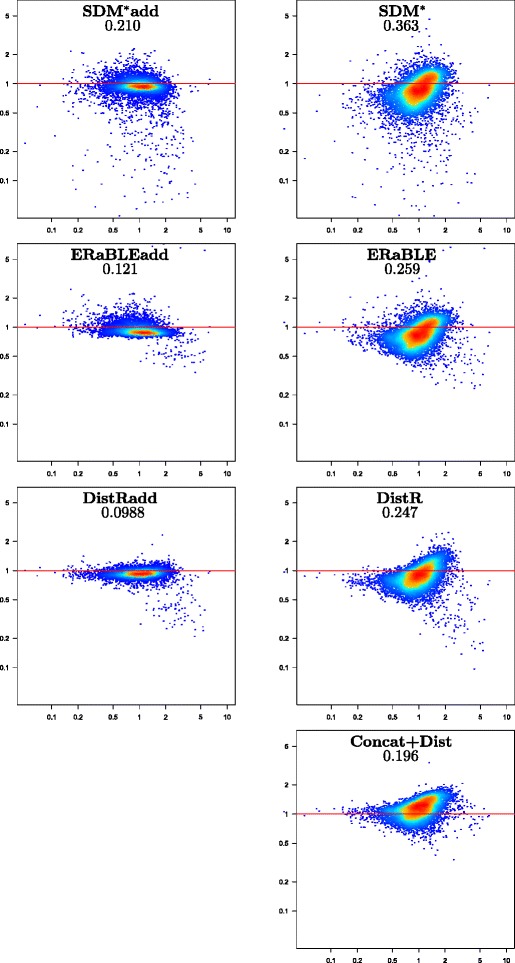
Estimation accuracy for gene rates in the OrthoMaM data set. Logarithmic scatterplots showing the 6,953 “reference” gene rates $\hat {r}^{ML}_{k}$ estimated by Concat+ML (x-axis), against ratios $\hat {r}_{k}/\hat {r}^{ML}_{k}$ (y-axis). Note that errors relative to the reference gene rates are measured with ratios, instead of differences. Colors (from blue to red) indicate increased density of points. The horizontal red line corresponds to no difference between the two estimates. Method names are shown at the top of each plot, followed by the mean absolute log-ratio between estimated and reference gene rates (see Additional file 3)

***Gene rate estimation.*** Figure 5 shows the accuracy of all tested methods in the estimation of gene rates. Two observations can be made: (1) the main difference in accuracy is now between supertree methods and all other methods (whereas on simulated data, the main difference was between SDM-based methods and the others); (2) again estimates are at slightly different scales, with supertree and medium-level methods having a tendency to estimate lower rates than Concat+ML.

Observation (1) is due to the use of a unique Gamma shape parameter (0.5), common to all genes, to estimate all matrices *Δ*_1_,*Δ*_2_,…,*Δ*_*m*_. Although this is common practice in distance-based analyses, for many genes this is far from the biological reality, as the shape parameters are themselves very different from gene to gene (the distribution of shape parameters inferred by PhyML has a 5 % quantile of 0.21, a median of 0.493, and a 95 % quantile of 1.73). Because distance estimates are monotonically decreasing functions of the shape parameter [47], underestimating (or overestimating) the shape parameter for gene *G*_*k*_ results in overestimating (respectively, underestimating) all the distances in *Δ*_*k*_, and therefore the rate *r*_*k*_. This explains the poor accuracy in gene rate estimation for all methods that use *Δ*_1_,*Δ*_2_,…,*Δ*_*m*_ (i.e., the medium-level methods and Concat+Dist).

It is possible to confirm this explanation by inspecting the genes corresponding to dots that significantly deviate from the red line in Fig. 5, which as expected tend to have ML Gamma shape parameters strongly deviating from 0.5 (not shown). Alternatively, Fig. 5 bis in Additional file 6 shows that if we use gene-specific Gamma shape parameters in the estimation of *Δ*_1_,*Δ*_2_,…,*Δ*_*m*_, then rate estimates become much more accurate for all methods that use these matrices. (However, note that this information is not available from pairwise sequence comparisons only). Figure 5 bis also shows that, once the effect described above is taken away, SDM-based methods become again the least accurate — consistent with our results for the simulated data set.

As for observation (2) — the fact that gene rate estimates tend to be lower than those of Concat+ML for all methods except Concat+Dist (see Fig. 5) — it is easy to understand that this is strictly linked to the fact that the estimated branches tend to be longer than those of Concat+ML for all methods except Concat+Dist (see Fig. 4).

***Discussion.*** One of the main differences with the results on the simulated data is the difficulty of setting a scale for branch lengths and gene rates. We expect this observation to extend to most real data sets, where inferring absolute estimates, rather than relative, may be very challenging.

Apart from this scaling issue, the results on the OrthoMaM data set are largely in line with those obtained on the simulated data set: DistR-based methods (DistR and DistRadd) lead to inaccurate branch length estimates, and SDM-based methods (SDM^*^ and SDM^*^add) lead to inaccurate gene rate estimates — which is not surprising, given that neither of these methods was originally designed for both these tasks (in fact SDM^*^ was designed for neither of these tasks). As for Concat+Dist, the high variance in branch length estimates observed on the simulated data seems to not be present for OrthoMaM. This is surprising, but we recall that it is hard to draw firm conclusions on estimation accuracy from this data set, for the reasons explained above.

That leaves us with ERaBLE-based methods and Concat+ML. It would seem that the choice among ERaBLE, ERaBLEadd and Concat+ML should largely be done based on their tradeoff between accuracy and computational cost (the first method being the fastest and the last the most accurate). One important lesson that the experiments on OrthoMaM highlight, however, is that unless we adopt gene-specific parameters modelling rates-across-sites heterogeneity (e.g., gene-specific Gamma shape parameters), medium-level methods such as ERaBLE may produce inaccurate gene rate estimates.

## Conclusion

In this paper we have examined the notions of branch lengths in a species tree and of gene rates in a phylogenomic context. We have presented ERaBLE, a novel and efficient method for the estimation of these quantities, which are often overlooked in phylogenomic analyses – for example by classical supertree methods – or whose estimation requires computationally-demanding methodologies – usually likelihood-based analysis of a concatenated superalignment. Note that for large phylogenomic data sets such as OrthoMaM – where the concatenated alignment consists of more than 6 million sites – the application of likelihood is very onerous, especially in terms of memory requirements, which may be problematic for some users. Recall that in our experiments we have constrained the tree topology; a full likelihood analysis would further increase computational costs. Moreover, in Additional file 7, we show that ML methods more efficient than PhyML – namely ExaML [52] and FastTree 2 [53] – are still very inefficient relative to ERaBLE.

Methodologically, ERaBLE represents the fastest available method to estimate the branch lengths of a given topology from a collection of distance matrices – one matrix per gene under consideration. It generalises and reduces to (when only one matrix is provided) classical WLS branch length estimation. The most important difference with single-gene WLS is that ERaBLE also estimates gene rate parameters, modelling the different “speeds” of evolution of different genes — with little computational overhead.

ERaBLE’s limitations are its reliance on a tree topology – either a well-accepted phylogeny or a tree reconstructed prior to its execution – and its seemingly strong assumptions about the data (orthology of the genes under analysis, and the proportional model). However, we stress that these hypotheses represent an ideal scenario. As shown by the experiments on the OrthoMaM data set, ERaBLE can perform well on real-world data sets where these assumptions will probably be violated to some degree, namely because of phenomena such as heterotachy [37] or limited topological incompatibilities due to incomplete lineage sorting (ILS), gene duplication and/or lateral gene transfer [35]. In order to investigate the robustness of ERaBLE and competing methods to these violations, it would be interesting to simulate data following more realistic assumptions, for example those of the multispecies coalescent [33] to study the effects of ILS. Furthermore, it would be useful to model alignment errors which are undoubtedly present in real data.

ERaBLE can be used in two ways, which differ in the way the input distance matrices are obtained: they can either be directly estimated from gene alignments, or they can be based on phylogenetic trees inferred for each gene. Our experiments (“Results and discussion” section) show that both these approaches provide valid alternatives to existing methodologies: the alternative methods are either only accurate for one of the two tasks that ERaBLE carries out — SDM-based methods provide branch lengths estimates comparable to those of ERaBLE, while DistR-based methods provides marginally better gene rates estimates — or computationally very demanding — as in the case of ML analysis of a concatenated superalignment.

A possible use of ERaBLE is as a complement to classical supertree methods (e.g., MRP [20, 21]), which often disregard branch length information, yet present in the input trees. In this context, ERaBLE would allow to rapidly assign meaningful branch lengths to the tree topologies reconstructed by these methods.

Alternatively, when (most of) the evolutionary relationships between the species under consideration are relatively well-known, ERaBLE can be used as a standalone, using a reference topology as input. This is the scenario that we have assumed in our experiments on the OrthoMaM data set. An interesting question for future research is the robustness of ERaBLE’s estimates to errors in the reference topology.

Furthermore, it would be interesting to investigate the possibility of combining the assignment of branch lengths made by ERaBLE with a criterion for topological inference, allowing to score different tree topologies for their fit with the data. This is analogous to what is done in classical distance-based phylogenetics, where least squares branch lengths can be used in combination with criteria such as minimum evolution [54]. However, this would probably need more methodological advances, first, to reduce further the time needed to evaluate a topology — if possible, by extending the approaches shown for particular cases of WLS in recent studies [40, 43, 55] — and, second, to avoid issues of statistical inconsistency, which are known to affect single-matrix WLS in combination with minimum evolution [56].

## Availability of supporting data

The simulated data set and the OrthoMaM data set supporting the results of this article can be found online at: http://www.atgc-montpellier.fr/erable.

## Additional files

Additional file 1
**ERaBLE, in detail.** (PDF 261 kb)

Additional file 2
**The choice of** ***Z***_***k***_ **in the constraint.** (PDF 514 kb)

Additional file 3
**Estimation accuracy measures.** (PDF 233 kb)

Additional file 4
**The scales of branch lengths for the OrthoMaM data set.** (PDF 359 kb)

Additional file 5
**Reference topology for the OrthoMaM data set.** (PDF 316 kb)

Additional file 6
**Usefulness of gene-specific Gamma shape parameters.** (PDF 1413 kb)

Additional file 7
**Alternative ML methods for Concat+ML.** (PDF 1443 kb)

## Abbreviations

ACS: Average Consensus Supertree
BWD: Build With Distances
ERaBLE: Evolutionary Rates and Branch Length Estimation
ML: Maximum Likelihood
MRP: Matrix Representation with Parsimony
SDM: Super Distance Matrix
OLS: Ordinary Least Squares
WLS: Weighted Least Squares
GLS: Generalized Least Square
