# Supplementary material for: Fast and accurate branch lengths estimation for phylogenomic trees
Source: BMC Bioinformatics. 2016 Jan 7;17:23. doi: 10.1186/s12859-015-0821-8 (PMC4705742; doi:10.1186/s12859-015-0821-8)
Supplement: Additional file 1 — ERaBLE, in detail. (PDF 261 kb) [file 12859_2015_821_MOESM1_ESM.pdf]

Additional file 1 — ERaBLE, in detail

In this additional file we show how ERaBLE computes the solution for problem (6). We start by introducing some notation that allows us to rewrite the problem in matrix form.

Inputs and outputs. First, let $\hat{\alpha} = (\hat{\alpha}_1, \hat{\alpha}_2, \ldots, \hat{\alpha}_m)^t$ and $\hat{b} = (\hat{b}_1, \hat{b}_2, \ldots, \hat{b}_\tau)^t$ designate the unknowns of the problem in column vector form, where $\tau = |E(\mathcal{T})|$ is the number of branches of the topology $\mathcal{T}$ and the superscript t denotes the transpose operator. Let then δ_k be a vector listing all the input distances $\delta_{ij}^{(k)}$ for gene G_k in lexicographic order with respect to the taxon indices. For example, if $L_1 = \{1, 2, 4, 6\}$, then $\delta_1 = (\delta_{12}^{(1)}, \delta_{14}^{(1)}, \delta_{16}^{(1)}, \delta_{24}^{(1)}, \delta_{26}^{(1)}, \delta_{46}^{(1)})^t$. Similarly, let $\hat{d}$ denote the vector containing the additive distances $\hat{d}_{ij}$ resulting from the branch lengths in $\hat{b}$, again ordered lexicographically. Finally, let $\hat{d}_k$ be the vector that is obtained from $\hat{d}$ by removing all the distances involving taxa not in L_k. Informally, problem (6) requires the vectors $\hat{\alpha}_k \delta_k$ and $\hat{d}_k$ to be as close as possible, for all $k \in \{1, 2, \ldots, m\}$.

Topological matrices. Now, let A be the topological matrix representing $\mathcal{T}$. This is a $n(n-1)/2 \times \tau$ binary matrix, whose τ columns correspond to branches of $\mathcal{T}$, and whose $n(n-1)/2$ rows correspond to pairs of taxa in L, in lexicographical order (see, e.g., [42]). $A = (a_{ij,e})$ is defined by setting $a_{ij,e} = 1$, if e is on the path between i and j, and 0 otherwise. Moreover, let A_k be the $|L_k|(|L_k| - 1)/2 \times \tau$ binary matrix that is obtained from A by removing all the rows corresponding to taxa not in L_k. Using these notations, we can write $\hat{d} = A\hat{b}$, and $\hat{d}_k = A_k \hat{b}$.

Weight matrices and vectors. Let W_k be the square matrix of order $|L_k|(|L_k|-1)/2$ whose diagonal entries are the weights $w_{ij}^{(k)}$, and whose every other element is zero. Finally, let $z = (Z_1, Z_2, \ldots, Z_m)^t$ and Z denote the sum of all Z_k, for $k = 1, 2, \ldots, m$.

The problem and its resolution via Lagrange multipliers. Using the notation above, problem (6) can be expressed as follows:

$$\begin{cases} \text{minimize}_{\hat{\alpha}, \hat{b}} & Q(\hat{\alpha}, \hat{b}) = \sum_{k=1}^{m} (\hat{\alpha}_k \delta_k - A_k \hat{b})^t W_k (\hat{\alpha}_k \delta_k - A_k \hat{b}), \\ \text{subject to} & z^t \hat{\alpha} = Z. \end{cases} \tag{8}$$

We solve problem (8) using the method of Lagrange multipliers [41]. This method relies on the Lagrangian function, which here is given by $\mathcal{L}(\hat{\alpha}, \hat{b}, \lambda) = Q(\hat{\alpha}, \hat{b}) + \lambda(z^t \hat{\alpha} - Z)$. A necessary condition for $(\hat{\alpha}, \hat{b})$ to be a solution of problem (8) is that all the partial derivatives of $\mathcal{L}$ be zero:

$$\nabla_{\hat{\alpha}, \hat{b}, \lambda} \mathcal{L}(\hat{\alpha}, \hat{b}, \lambda) = 0. \tag{9}$$

Although in general (9) is only a necessary condition for a minimum, here it is also sufficient, as the function to minimize $Q(\hat{\alpha}, \hat{b})$ is a sum of squares, thus convex, and the constraint is linear. Solving problem (8) is thus equivalent to solving equation (9), which can be written as follows:

$$\begin{cases} \dfrac{\partial \mathcal{L}}{\partial \hat{\alpha}_k} = 0 & \Leftrightarrow & \hat{\alpha}_k \delta_k^t W_k \delta_k - \delta_k^t W_k A_k \hat{b} + Z_k \lambda / 2 = 0 & m \text{ equations } (k = 1, \ldots, m). \\ \nabla_{\hat{b}} \mathcal{L} = 0 & \Leftrightarrow & \sum_{k=1}^{m} \left(A_k^t W_k A_k \hat{b} - \hat{\alpha}_k A_k^t W_k \delta_k \right) = 0 & \tau \text{ equations.} \\ \dfrac{\partial \mathcal{L}}{\partial \lambda} = 0 & \Leftrightarrow & z^t \hat{\alpha} = Z & 1 \text{ equation.} \end{cases} \tag{10}$$

To simplify system (10) we define the following matrices: D is the $m \times m$ matrix whose diagonal entries are the scalars $\delta_k^t W_k \delta_k$, and whose every other element is zero; B is the $\tau \times m$ matrix whose columns are the vectors $-A_k^t W_k \delta_k$; C is the $\tau \times \tau$ matrix defined by $C = \sum_{k=1}^{m} A_k^t W_k A_k$. After dropping the 1/2 coefficient for λ, as we are not interested in the value of the multiplier, system (10) can be written

as:

$$\begin{cases} D\hat{\alpha} + B^t\hat{b} + \lambda z = 0, & (11) \\ B\hat{\alpha} + C\hat{b} = 0, & (12) \\ z^t\hat{\alpha} = Z. & (13) \end{cases}$$

Naïve matrix multiplication allows to calculate the coefficients of this system in $\mathcal{O}(mn^4)$ time, as this is dominated by the computation of $C = \sum_k A_k^t W_k A_k$, where each $A_k^t W_k A_k$ can be obtained in $\mathcal{O}(\tau^2 n^2) = \mathcal{O}(n^4)$ time (using the fact that W_k is diagonal). Adding to this the time taken by standard algorithms for the resolution of this system in $\mathcal{O}(m+n)$ equations and unknowns, we get to a total complexity of $\mathcal{O}(mn^4 + (n+m)^3)$ time for the naïve algorithm. For the data sets typical in phylogenomics this would be unfeasible. Below, we show how to bring this down to $\mathcal{O}(mn^2 + n^3)$ time.

Efficient solution of the linear system. First isolate $\hat{\alpha}$ in (11):

$$\hat{\alpha} = -D^{-1}\big(B^t\hat{b} + \lambda z\big). \tag{14}$$

Then substitute $\hat{\alpha}$ in (13) and isolate λ:

$$\lambda = -\frac{Z + z^t D^{-1} B^t \hat{b}}{z^t D^{-1} z} = -\frac{Z + u^t\hat{b}}{\omega},$$

where we define the vector $u = BD^{-1}z$ and the scalar $\omega = z^t D^{-1} z$. Replace then λ in (14) with the expression just obtained:

$$\hat{\alpha} = D^{-1}\Big(-B^t\hat{b} + z\Big(\frac{Z + u^t\hat{b}}{\omega}\Big)\Big) = D^{-1}\Big(\frac{zu^t}{\omega} - B^t\Big)\hat{b} + \frac{Z}{\omega}D^{-1}z. \tag{15}$$

Then replace $\hat{\alpha}$ with expression (15) in equation (12):

$$0 = B\hat{\alpha} + C\hat{b} = \Big(C + \frac{uu^t}{\omega} - BD^{-1}B^t\Big)\hat{b} + \frac{Z}{\omega}u.$$

If we let

$$M = \Big(C + \frac{uu^t}{\omega} - BD^{-1}B^t\Big),$$

then $\hat{b}$ can be found by solving the following system:

$$M\hat{b} = -\frac{Z}{\omega}u. \tag{16}$$

Finally $\hat{\alpha}$ can be obtained by using the value found for $\hat{b}$ in (15).

Remark on the scale of the results. Equations (16) and (15) — whose right-hand-sides are directly proportional to Z — show that the solutions $\hat{\alpha}$ and $\hat{b}$ scale proportionally with Z, the right-hand side of the constraint in our problem (8). Since ERaBLE subsequently resets the scale of $\hat{\alpha}$ and $\hat{b}$, by multiplying them by the correction factor in equation (7), this shows that the value of Z is irrelevant to the end results.

Uniqueness of the solution. If M is not invertible, then our optimization problem has multiple solutions. This happens when the sequence coverage is insufficient, with pairs of taxa i, j in crucial positions within $\mathcal{T}$, such that $\delta_{ij}^{(k)}$ is undefined for all $k \in \{1, 2, \ldots, m\}$. We note however that for the data sets that we consider here — with at least hundreds of genes — it is very unlikely to encounter this problem, unless the sequence coverage is extremely low. For example, the solution is unique for all the data sets we used in our experiments (simulated or real, and whose matrices cover from 4 to 40 taxa). A precise mathematical characterisation of the data sets guaranteeing the uniqueness of solutions is possible, but beyond the scope of this paper.

Computational complexity. M is a square matrix of order $\tau = \mathcal{O}(n)$. The resolution of the linear system in equation (16) can be carried out using standard algorithms in $\mathcal{O}(n^3)$ time and $\mathcal{O}(n^2)$ memory.

Taking into account all the other operations involved — most notably calculating all the coefficients of this linear system — the total complexity of ERaBLE is then of $\mathcal{O}(n^3 + mn^2)$ time and $\mathcal{O}(n^2 + mn)$ memory (in addition to that used to store the inputs). This is dominated by the computation of the entries in the matrices B, C, D, as we now show.

Computing matrix B. Bryant and Waddell [42] showed that it is possible to calculate the product $A^t v$, where A is a topological matrix for a tree with n leaves, and v is any vector with $n(n-1)/2$ entries, with a time complexity of $\mathcal{O}(n^2)$. This algorithm is trivially generalized to a partial topological matrix A_k, meaning that we can calculate each column of B, that is $-A_k^t(W_k\delta_k)$, in $\mathcal{O}(n^2)$ time, leading to a total time complexity of $\mathcal{O}(mn^2)$ for calculating B. Note that storing B requires $\mathcal{O}(mn)$ memory.

Computing matrix C. Recall that $C = \sum_{k=1}^m A_k^t W_k A_k$. We show that each each $A_k^t W_k A_k$ can be computed in $\mathcal{O}(n^2)$ time, leading to a time complexity of $\mathcal{O}(mn^2)$ for calculating C.

$A_k^t W_k A_k$ is a $\tau \times \tau$ square matrix where each entry corresponds to a pair of branches e, f in $\mathcal{T}$. It is easy to see that the entries of this matrix can be expressed as follows:

$$(A_k^t W_k A_k)_{ef} = \sum_{\substack{i \in X \cap L_k \\ j \in Y \cap L_k}} w_{ij}^{(k)}, \tag{17}$$

where X and Y denote the disjoint sets of taxa separated by both e and f, as shown in Fig. 6. (Formally, X and Y are the disjoint sets of taxa such that any path from an element of X to an element of Y must pass via both e and f.)

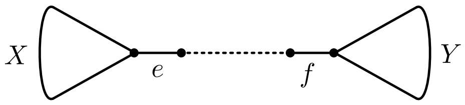

Figure 6 – X and Y are the disjoint sets of taxa separated by both e and f.

Now let $C_{XY}^{(k)}$ denote the right-hand side of Eqn. (17). We can calculate all the $C_{XY}^{(k)}$ values recursively:

– If X and Y are singletons with $X = \{i\}$ and $Y = \{j\}$, then :

$$C_{XY}^{(k)} = C_{ij}^{(k)} = \begin{cases} w_{ij}^{(k)} & \text{if } \{i,j\} \subset L_k, \\ 0 & \text{otherwise.} \end{cases}$$

– Otherwise, one of the two taxon sets, say, Y can be decomposed in a number of disjoint subsets $Y = \bigcup_{i=1}^d Y_i$, corresponding to the subtrees of the tree rooted in f and having Y as leaf set. Then:

$$C_{XY}^{(k)} = \sum_{i=1}^{d} C_{XY_i}^{(k)}.$$

Since there are $\mathcal{O}(n^2)$ $C_{XY}^{(k)}$ values to calculate, the entire matrix $A_k^t W_k A_k$ can be filled in $\mathcal{O}(n^2)$ time and requires $\mathcal{O}(n^2)$ memory to be stored.

Computing matrix D. Since D is a diagonal matrix, we only need to calculate and store the elements on its diagonal, each of which can be trivially obtained in $O(n^2)$ time, leading to a total of $O(mn^2)$ time and $O(m)$ memory.

Other computations. All the remaining calculations can be done within complexities that are of the same order as, or inferior to those detailed above. Thus $\hat{b}$ and $\hat{\alpha}$ can be obtained in $\mathcal{O}(n^3 + mn^2)$ time and $\mathcal{O}(n^2 + mn)$ (auxiliary) memory.
