# Supplementary material for: Fast and accurate branch lengths estimation for phylogenomic trees
Source: BMC Bioinformatics. 2016 Jan 7;17:23. doi: 10.1186/s12859-015-0821-8 (PMC4705742; doi:10.1186/s12859-015-0821-8)
Supplement: Additional file 2 — The choice of Z k in the constraint. (PDF 514 kb) [file 12859_2015_821_MOESM2_ESM.pdf]

Additional file 2 — The choice of Z_k in the constraint

While testing ERaBLE we have realised that setting $Z_k = 1$ or $Z_k = N_k$, for all $k \in \{1, 2, \ldots, m\}$ can cause important overestimations of the scale factors $\hat{\alpha}_k$ for genes only present in a small group of closely related taxa. This phenomenon is strictly linked to the strong underestimation of a minority of gene rates — and the slight overestimation of the majority of gene rates — observed for SDM-based methods, which also use the constraint with $Z_k = 1$. In our experiments we have set $Z_k = N_k \sum_{i,j \in L_k} \delta_{ij}^{(k)}$, which largely solves this problem, despite being rather heuristic. In this additional file, we show the importance of the constraint used by ERaBLE with a very simple example.

We construct a small data set consisting of just two nucleotide alignments, those of exons ENSG000000 66654_THUMPD1_000 and ENSG00000127423_AUNIP_000 obtained from OrthoMaM after trimAl filtering. We call them G_1 and G_2, respectively. For simplicity we only keep the sequences of six species, those in the set $L = \{$*Gorilla, Homo, Pan, Bos, Erinaceus, Sorex*$\}$. Since G_1 is only sampled in primates, we have $L_1 = \{$*Gorilla, Homo, Pan*$\}$, and $L_2 = L$. Alignment lengths are $N_1 = 489$ for G_1, and $N_2 = 855$ for G_2. Figure 7 shows a phylogenetic tree for these data.

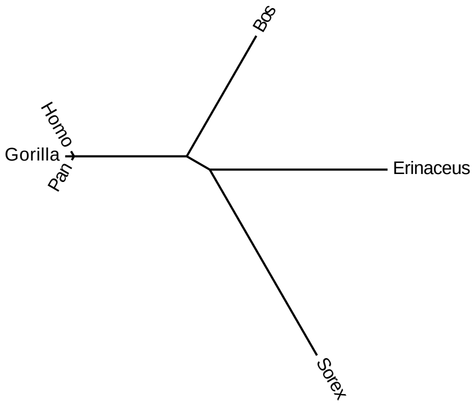

Figure 7 – Maximum likelihood tree (PhyML with model TN93+Γ_8) obtained on the concatenation of the two alignments in the example.

Fig. 8 shows the distance matrices estimated for G_1 and G_2 (left column), and the different behaviours of ERaBLE with $Z_k = 1$ and $Z_k = N_k \sum_{i,j \in L_k} \delta_{ij}^{(k)}$ (middle and right column, respectively). The behaviour for $Z_k = N_k$ is similar to that for $Z_k = 1$, and not shown here for brevity. A quick comparison of Δ_1 and Δ_2 suggests that the rate of G_1 is higher than that of G_2 (note that, in two cases out of three, $\delta_{ij}^{(1)}$ is more than the double than $\delta_{ij}^{(2)}$). We then expect that $\hat{\alpha}_1 < \hat{\alpha}_2$. However, when $Z_k = 1$, the opposite happens: solving problem (6) leads to $\hat{\alpha}_1 = 1.73$ and $\hat{\alpha}_2 = 0.274$.

The key observation to understand why this happens is that Δ_1 only contains very closely related species (all great apes, see also Fig. 7), so its distances are very small relative to many of those in Δ_2, which can be up to about 30 times larger. As a consequence, if $\hat{\alpha}_1 \leq \hat{\alpha}_2$ the value of the objective function $Q(\hat{\alpha}, \hat{b})$ is dominated by the differences $\hat{\alpha}_2 \delta_{ij}^{(2)} - \hat{d}_{ij}$, that is, the difference between $\hat{\alpha}_2 \Delta_2$ and $\widehat{D} = (\hat{d}_{ij})$. It is then intuitive that a way to reduce $Q(\hat{\alpha}, \hat{b})$ is to simultaneously reduce the scale of $\hat{\alpha}_2 \Delta_2$ and $\widehat{D} = (\hat{d}_{ij})$, which can be achieved by decreasing the value of $\hat{\alpha}_2$ (and consequently increasing that of $\hat{\alpha}_1$, given that for $Z_k = 1$ their mean is constrained to be 1).

This is precisely what is happening when setting $Z_k = 1$ in our example (middle column in Fig. 8): instead of having $\hat{\alpha}_1 < \hat{\alpha}_2$, ERaBLE produces a small $\hat{\alpha}_2 = 0.274$ and a large $\hat{\alpha}_1 = 1.73$. Compared to the alternative constraint (right column), where we have $\hat{\alpha}_1 < \hat{\alpha}_2$ as expected, it is clear that this results

Input distances

Δ_1

	Homo	Pan
Gorilla	.0203	.0135
Homo		.0148

Δ_2

	Homo	Pan	Bos	Erinac.	Sorex
Gorilla	.0087	.0099	.278	.354	.432
Homo		.0062	.279	.342	.432
Pan			.286	.345	.429
Bos				.419	.422
Erinac.					.446

Trivial constraint $Z_k = 1$

$\hat{\alpha}_1$	$\hat{\alpha}_2$
1.73	.274

$\hat{\alpha}_1\Delta_1$

	Homo	Pan
Gorilla	.0351	.0233
Homo		.0256

$\hat{\alpha}_2\Delta_2$

	Homo	Pan	Bos	Erinac.	Sorex
Gorilla	.0024	.0028	.0763	.0970	.118
Homo		.0017	.0766	.0937	.118
Pan			.0783	.0945	.118
Bos				.115	.116
Erinac.					.122

$\widehat{D}$

	Homo	Pan	Bos	Erinac.	Sorex
Gorilla	.0127	.0117	.0777	.0985	.116
Homo		.0104	.0774	.0983	.116
Pan			.0764	.0973	.115
Bos				.107	.124
Erinac.					.122

Our constraint $Z_k = N_k \sum_{i,j \in L_k} \delta_{ij}^{(k)}$

$\hat{\alpha}_1$	$\hat{\alpha}_2$
0.538	1.00

$\hat{\alpha}_1\Delta_1$

	Homo	Pan
Gorilla	.0109	.0073
Homo		.0080

$\hat{\alpha}_2\Delta_2$

	Homo	Pan	Bos	Erinac.	Sorex
Gorilla	.0087	.0099	.278	.354	.432
Homo		.0062	.279	.342	.432
Pan			.286	.345	.429
Bos				.419	.422
Erinac.					.446

$\widehat{D}$

	Homo	Pan	Bos	Erinac.	Sorex
Gorilla	.0087	.0099	.284	.360	.424
Homo		.0069	.280	.357	.420
Pan			.282	.358	.422
Bos				.390	.454
Erinac.					.447

Figure 8 – **Changing behaviour of ERaBLE with different contraints.** In this example, ERaBLE is run on the two distance matrices Δ_1 and Δ_2 on the left. Setting $Z_k = 1$ results in the $\hat{\alpha}_k$ values and matrices $\hat{\alpha}_1\Delta_1, \hat{\alpha}_2\Delta_2$ and $\widehat{D} = (\hat{d}_{ij})$ in the middle column. Our chosen setting for Z_k results in more reasonable values for these quantities (right column), as explained in the text.

in significantly smaller differences $\hat{\alpha}_2\delta_{ij}^{(2)} - \hat{d}_{ij}$ for most distances, the only exceptions being the three distances between primates. Note that the scale of $\hat{\alpha}_2\Delta_2$ cannot be reduced indefinitely, as then the scale of $\hat{\alpha}_1\Delta_1$ becomes too large, and the fit of $\widehat{D}$ with the distances between primates in the two rescaled distance matrices becomes too loose (note that for $Z_k = 1$ the distances between primates in $\hat{\alpha}_1\Delta_1$ and $\hat{\alpha}_2\Delta_2$ already differ by an order of magnitude and the fit with $\widehat{D}$ is very poor).

The, admittedly heuristic, approach that we have adopted in our experiments, that is, setting $Z_k = N_k \sum_{i,j \in L_k} \delta_{ij}^{(k)}$, essentially prevents the genes only appearing in few and closely related taxa from having an influence on the constraint. Thus, it is the $\hat{\alpha}_k$ for the remaining genes that are constrained to have a weighted average of 1 (where the weight depends on the length of their sequence, as in equation (5)). As a consequence, these $\hat{\alpha}_k$ cannot be reduced together with $\widehat{D}$, as we showed for $Z_k = 1$. In our example, the new constraint is $24 \cdot \hat{\alpha}_1 + 3838 \cdot \hat{\alpha}_2 = 3862$, which is roughly equivalent to imposing $\hat{\alpha}_2 = 1$. The results are then much more realistic than with $Z_k = 1$: for example the rescaled matrices $\hat{\alpha}_1\Delta_1$ and $\hat{\alpha}_2\Delta_2$ are now much closer on their common entries (right column in Fig. 8).
