# Supplementary material for: Fast and accurate branch lengths estimation for phylogenomic trees
Source: BMC Bioinformatics. 2016 Jan 7;17:23. doi: 10.1186/s12859-015-0821-8 (PMC4705742; doi:10.1186/s12859-015-0821-8)
Supplement: Additional file 3 — Estimation accuracy measures. (PDF 233 kb) [file 12859_2015_821_MOESM3_ESM.pdf]

Additional file 3 — Estimation accuracy measures

We adopted two different measures of estimation accuracy, one for branch lengths and the other for gene rates.

– The fraction of variance unexplained is a classical measure in regression analysis which can be seen as a normalised form of the sum of squared errors of prediction of an estimation model. Here, we adapt it to measure the discrepancy between a vector of branch lengths (b_e) and their estimates $(\hat{b}_e)$. We define the *fraction of variance unexplained* of (b_e) relative to $(\hat{b}_e)$ as:

$$\frac{\sum_e (b_e - \hat{b}_e)^2}{\sum_e (b_e - \bar{b})^2}$$

where sums are over all branches in a tree and $\bar{b}$ is the arithmetic mean of the branch lengths in (b_e).

– In order to measure the discrepancy between a collection of m gene rates (r_k) and their estimates $(\hat{r}_k)$, we take the *mean absolute log-ratio* between r_k and $\hat{r}_k$, that is:

$$\frac{1}{m} \sum_{k=1}^{m} \left| \log \frac{\hat{r}_k}{r_k} \right| .$$
