# Supplementary material for: Fast and accurate branch lengths estimation for phylogenomic trees
Source: BMC Bioinformatics. 2016 Jan 7;17:23. doi: 10.1186/s12859-015-0821-8 (PMC4705742; doi:10.1186/s12859-015-0821-8)
Supplement: Additional file 4 — The scales of branch lengths for the OrthoMaM data set. (PDF 359 kb) [file 12859_2015_821_MOESM4_ESM.pdf]

Additional file 4 — The scales of branch lengths for the OrthoMaM data set

The OrthoMaM data set displays an inverse correlation between the rate r_k of a gene and the depth of its alignment (its "coverage", i.e., $|L_k|$ in our notation), as is clearly shown in Fig. 9. This is not surprising — it is expected that genes evolving more slowly are easier to sample and annotate in many taxa — and we thus expect most real data sets to display the same correlation, to varying degrees.

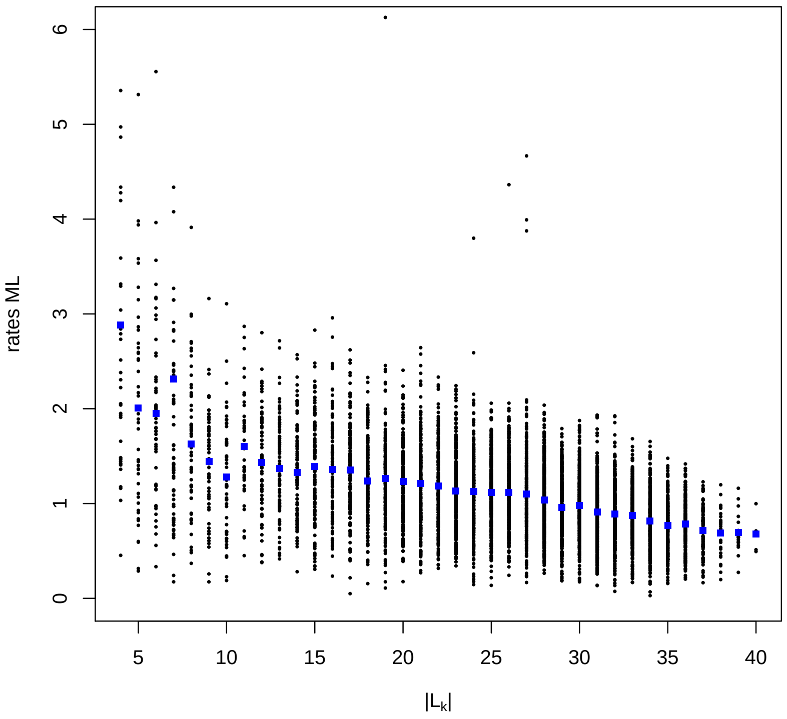

Figure 9 – **Correlation between the estimated rate of a gene and its alignment depth in the OrthoMaM data set.** For each of the 6,953 genes in the OrthoMaM data set, the number of sequences in its alignment (i.e., $|L_k|$, x-axis) is plotted against the rate estimate $\hat{r}_k$ produced by Concat+ML (y-axis). The blue squares represent the means of $\hat{r}_k$, for all genes with a fixed value of $|L_k| \in \{4, 5, \ldots, 40\}$.

This, however, poses a problem regarding the scale of the results. In loose terms, the problem is the following: *all other things being equal, should genes with high coverage influence more the scale of branch length estimates than genes with low coverage?* Note that the answer to this question only becomes relevant in data sets, such as OrthoMaM, where there is a correlation between coverage and rates: if, as realistic, genes sampled in a greater number of taxa tend to have lower rates, then answering *yes* to this question will result in shorter branch length estimates, than methods which implicitly answer *no* to it.

Close inspection of the methods in our experiments reveals that the answer to this question is *no* for the supertree and medium-level methods we tested, and *yes* for the superalignment methods. For ERaBLE-based and SDM-based methods, this is caused by the rescaling they apply to their estimates, which sets a scale that is determined by all genes in proportion to their lengths (Eqn. (5)), but which is independent of gene coverage.

As a result, the superalignment methods we tested tend to produce shorter branch length estimates than the supertree and medium-level methods we tested, which is precisely what we observe in Fig. 4. This explanation can also be confirmed by simulating data with an inverse correlation between $|L_k|$ and r_k, where similar differences in scales between the methods tested can be observed (not shown).
