# Supplementary material for: Fast and accurate branch lengths estimation for phylogenomic trees
Source: BMC Bioinformatics. 2016 Jan 7;17:23. doi: 10.1186/s12859-015-0821-8 (PMC4705742; doi:10.1186/s12859-015-0821-8)
Supplement: Additional file 5 — Reference topology for the OrthoMaM data set. (PDF 316 kb) [file 12859_2015_821_MOESM5_ESM.pdf]

Additional file 5 — Reference topology for the OrthoMaM data set

For the OrthoMaM data set, the reference topology follows the Atlantogenata hypothesis (e.g., [Morgan et al. 2013]) and the Laurasiatheria intra-order placements of Romiguier et al. [Romiguier et al. 2013]. In Newick format this topology is given by:

```
(Ornithorhynchus,((((((((((Mus,Rattus),Dipodomys),Cavia),Ictidomys),(Ochotona,Oryctolagus)),
(((Otolemur,Microcebus),(Tarsius,(Callithrix,(Macaca,(Nomascus,(Pongo,(Gorilla,(Pan,Homo)))
))))),Tupaia)),(((((Felis,(Canis,(Ailuropoda,Mustela))),Equus),(Myotis,Pteropus)),
(((Bos,Tursiops),Sus),Vicugna)),(Sorex,Erinaceus))),(((Loxodonta,Procavia),Echinops),
(Dasypus,Choloepus))),(Monodelphis,(Macropus,Sarcophilus))));
```

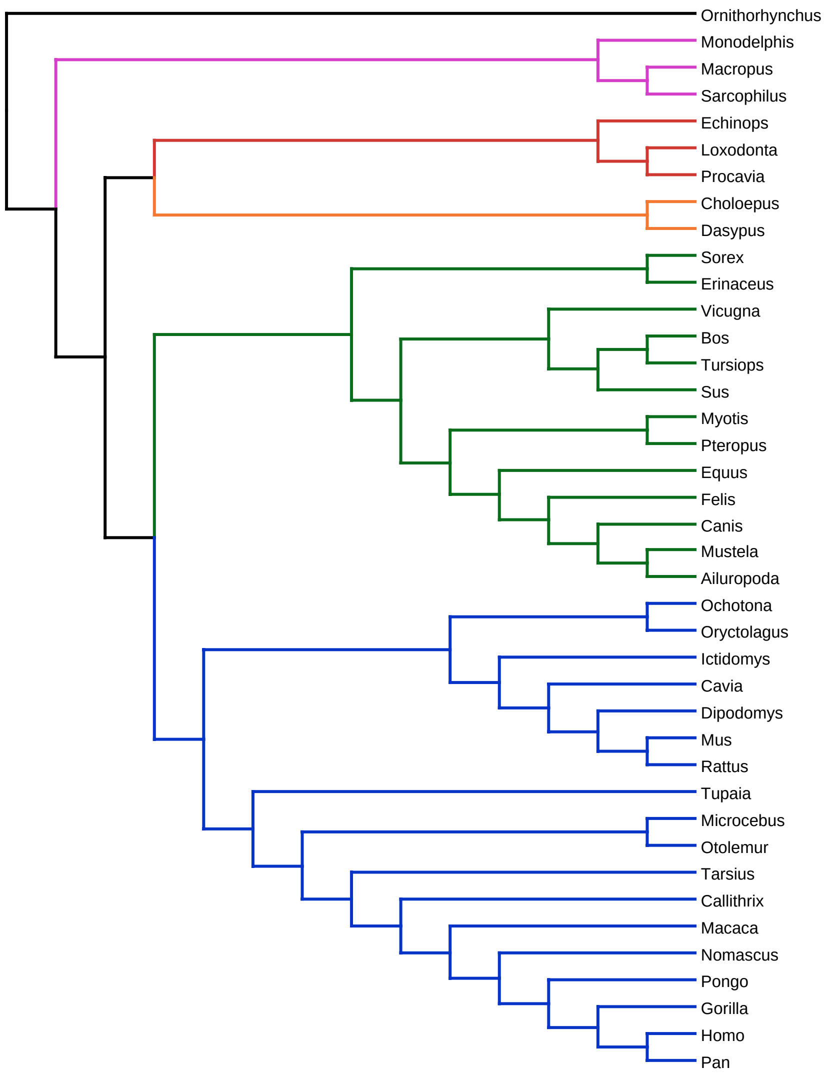

Figure 10 – **OrthoMaM data set reference topology.**

[Morgan et al. 2013] C. C. Morgan, P. G. Foster, A. E. Webb, D. Pisani, J. O. McInerney, and M. J. O'Connell, "Heterogeneous models place the root of the placental mammal phylogeny," *Molecular biology and evolution*, vol. 30, no. 9, pp. 2145–2156, 2013.

[Romiguier et al. 2013] J. Romiguier, V. Ranwez, F. Delsuc, N. Galtier, and E. J. Douzery, "Less is more in mammalian phylogenomics: At-rich genes minimize tree conflicts and unravel the root of placental mammals," *Molecular biology and evolution*, vol. 30, no. 9, pp. 2134–2144, 2013.
