# Supplementary material for: Fast and accurate branch lengths estimation for phylogenomic trees
Source: BMC Bioinformatics. 2016 Jan 7;17:23. doi: 10.1186/s12859-015-0821-8 (PMC4705742; doi:10.1186/s12859-015-0821-8)
Supplement: Additional file 6 — Usefulness of gene-specific Gamma shape parameters. (PDF 1413 kb) [file 12859_2015_821_MOESM6_ESM.pdf]

# Additional file 6 — Usefulness of gene-specific Gamma shape parameters

Here we show the results of repeating our experiments on the OrthoMaM data set with gene-specific Gamma shape parameters in the estimation of the distance matrices $\Delta_1, \Delta_2, \ldots, \Delta_m$ (namely, for $\Delta_k$ we use the shape parameter estimated by PhyML when inferring $\hat{T}_k$). Note that doing so has a positive effect on the accuracy of medium-level methods at estimating both branch lengths (Fig. 4 bis) and gene rates (Fig. 5 bis). In general such gene-specific Gamma shape parameters will not be available to users of medium-level approaches, as they cannot be inferred from pairwise sequence comparisons only. This is why our experiments set these parameters to a fixed value.

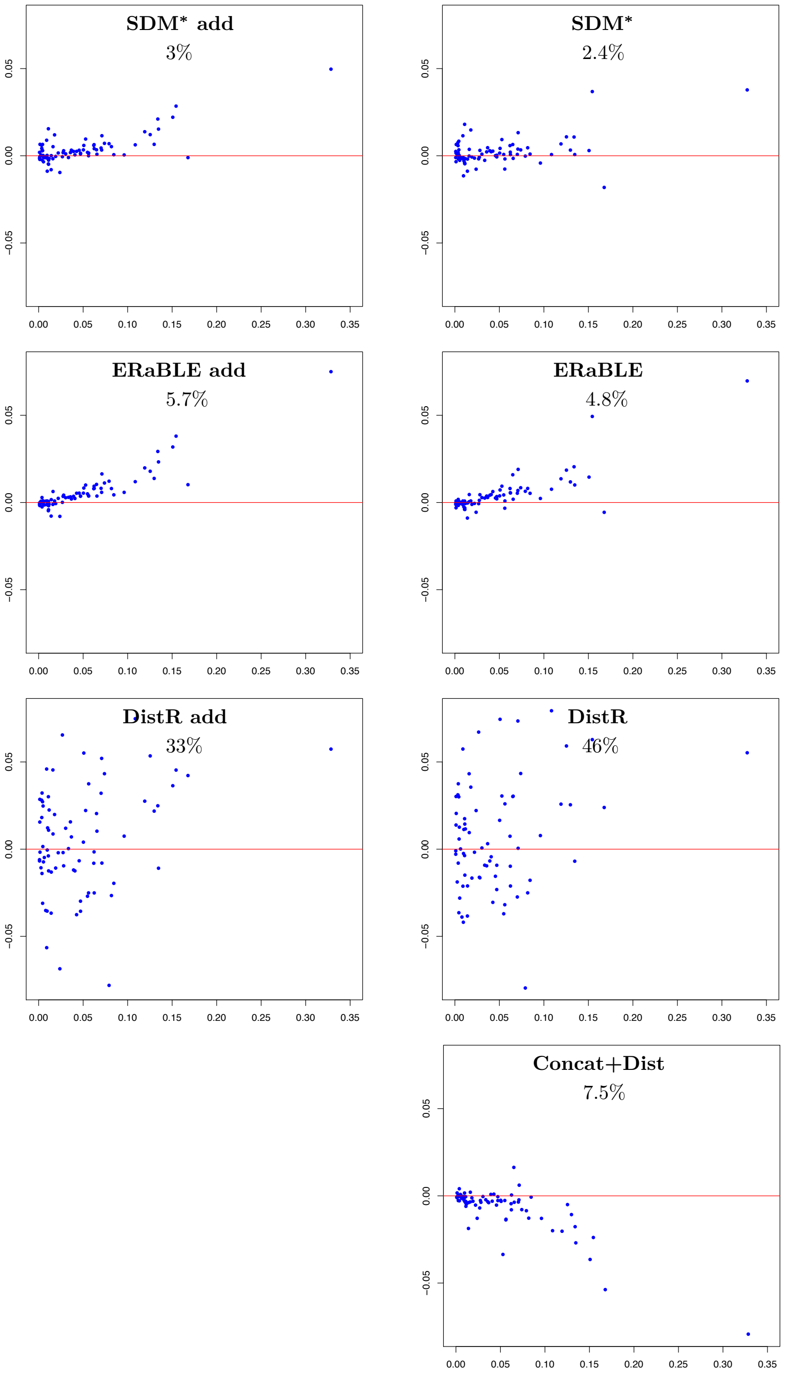


Figure 4 bis – **Accuracy of branch length estimates in the OrthoMaM data set.** Same as Fig. 4 in the main text, but here the Gamma shape parameter used for the estimation of $\Delta_k$ is set to the value estimated by PhyML when inferring $\hat{T}_k$. (Whereas in Fig. 4 the Gamma shape parameter is set to 0.5.)

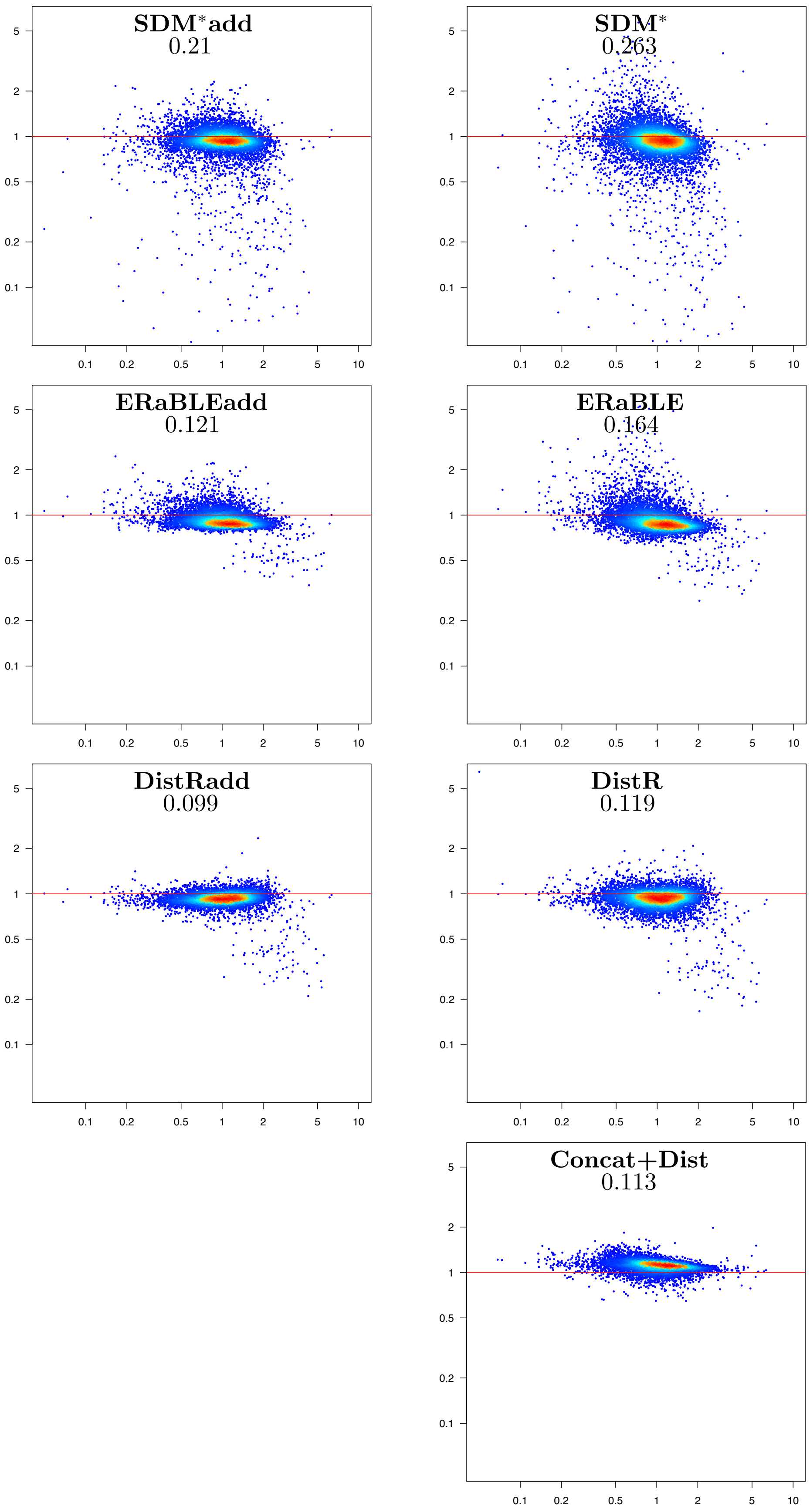


Figure 5 bis – **Estimation accuracy for gene rates in the OrthoMaM data set.** Same as Fig. 5 in the main text, but here the Gamma shape parameter used for the estimation of $\Delta_k$ is set to the value estimated by PhyML when inferring $\hat{T}_k$. (Whereas in Fig. 5 the Gamma shape parameter is set to 0.5.)
