# Supplementary material for: Fast and accurate branch lengths estimation for phylogenomic trees
Source: BMC Bioinformatics. 2016 Jan 7;17:23. doi: 10.1186/s12859-015-0821-8 (PMC4705742; doi:10.1186/s12859-015-0821-8)
Supplement: Additional file 7 — Alternative ML methods for Concat+ML. (PDF 1443 kb) [file 12859_2015_821_MOESM7_ESM.pdf]

Additional file 7 — Alternative ML methods for Concat+ML

Here we show the results for the estimation of branch length in the simulated data set and in the OrthoMaM data set with alternative ML methods for the pipeline Concat+ML. We recall that Concat+ML involves assigning branch lengths to the reference topology $\mathcal{T}$ by running topology-constrained PhyML on the superalignment (concatenate), with the model TN93+Γ_8. The PhyML alternatives we have considered are ExaML [52] and FastTree 2 [53]. These methods are more computationally efficient than PhyML, but support a narrower range of models of evolution. We ran ExaML with the GTR+Γ_4 model to assign branch lengths to the reference topology. The model and number of categories in the discrete Gamma distribution are not modifiable in ExaML. We call this pipeline Concat+ExaML. We ran FastTree 2 with the GTR+CAT model with the gamma option and call this pipeline Concat+FastTree. In both cases the topology is constrained to be $\mathcal{T}$. Fig. 4 ter shows the accuracy of these pipelines in the estimation of the branch lengths in the simulated data set and in the OrthoMaM data set. Table 3 gives their running times and memory usage.

In Fig. 4 ter, we observe that Concat+FastTree tends to overestimate short branch lengths and strongly underestimate long branch lengths. We cannot explain this bias at the moment. Concat+ExaML is slightly less accurate than Concat+ML in the estimation of branch lengths for the simulated data set. This may be explained by the different substitution model employed by Concat+ExaML. As expected, Concat+ExaML and Concat+FastTree methods have a reduced computational cost in time and memory in comparison with Concat+ML, but still relatively high, when compared to ERaBLE (Table 3).

Table 3 – **Computational efficiencies on the OrthoMaM data set for the tested methods.**

	**Concat+ML**	**Concat+ExaML**	**Concat+FastTree**	**ERaBLE**
Time	41h16m	14h20m	3h42m	7s
Memory	117 GB	15.4 GB	41.1 GB	221 MB

NOTE.— The first row gives the time to obtain estimates for branch lengths. The second row gives the maximum amount of memory allocated. All the experiments were conducted on a cluster machine with 200 GB RAM and a 2.66 GHz CPU because of the large memory requirements, except for ERaBLE whiwh was run on a standard PC with 4 GB RAM and a 2.7 GHz CPU.

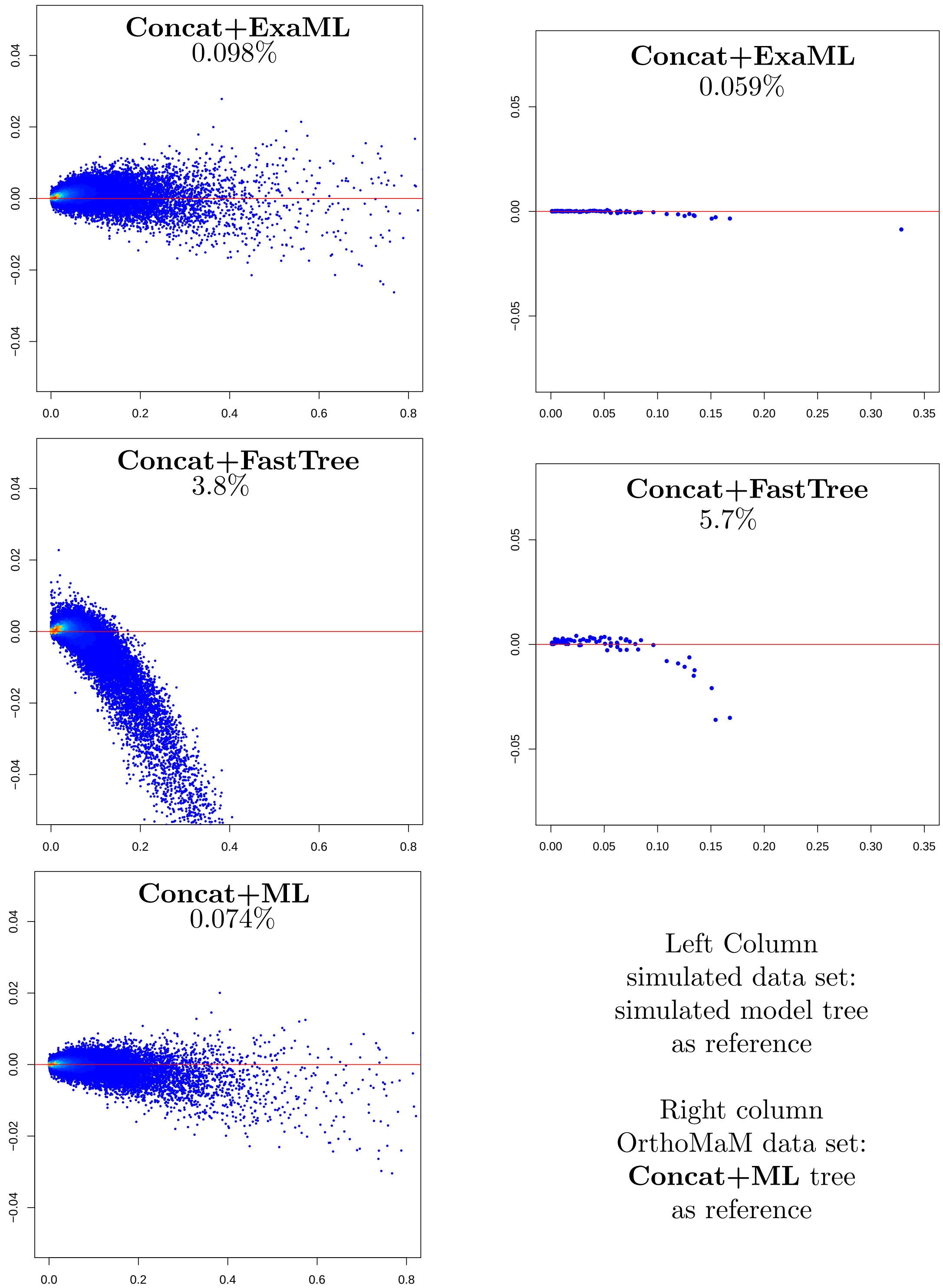

Figure 4 ter – **Accuracy of branch length estimates. Left column: accuracy for the simulated data set, right column: accuracy for the OrthoMaM data set.** For each method, the reference branch lengths b_e (x-axis) are plotted against the differences $\hat{b}_e - b_e$ (y-axis) (where $\hat{b}_e$ is the estimate for the length of e obtained by the method at the top of the plot). The horizontal red line corresponds to no difference between the two estimates. Method names are shown at the top of each plot, followed by the fraction of variance unexplained of (b_e) relative to $(\hat{b}_e)$. For the simulated data set, reference branch lengths are those of the 500 model trees. For the OrthoMaM data set, reference branch lengths are those estimated by Concat+ML on the reference topology. Colors (from blue to red) indicate increased density of points. Fore more detail, compare the left column with Fig. 2 and the right column with Fig. 4 in the main text.
